# Touch-evoked traveling waves establish a translaminar spacetime code

**DOI:** 10.1126/sciadv.adr4038

**Published:** 2025-01-31

**Authors:** Daniel L. Gonzales, Hammad F. Khan, Hayagreev V. S. Keri, Saumitra Yadav, Christopher Steward, Lyle E. Muller, Scott R. Pluta, Krishna Jayant

**Affiliations:** ^1^ Weldon School of Biomedical Engineering, Purdue University, West Lafayette, IN 47907, USA.; ^2^ Department of Biological Sciences, Purdue University, West Lafayette, IN 47907, USA.; ^3^ Department of Computer Science, Western University, London, ON, Canada.; ^4^ Department of Applied Mathematics, Western University, London, ON, Canada.; ^5^ Brain and Mind Institute, Western University, London, ON, Canada.; ^6^ Purdue Institute for Integrative Neuroscience, Purdue University, West Lafayette, IN 47907, USA.

## Abstract

Linking sensory-evoked traveling waves to underlying circuit patterns is critical to understanding the neural basis of sensory perception. To form this link, we performed simultaneous electrophysiology and two-photon calcium imaging through transparent NeuroGrids and mapped touch-evoked traveling waves and underlying microcircuit dynamics. In awake mice, both passive and active whisker touch elicited traveling waves within and across barrels, with a fast early component followed by a late wave that lasted hundreds of milliseconds poststimulus. Notably, late waves were modulated by perceived value and predicted behavioral choice in a two-whisker discrimination task. We found that the late wave feature was (i) modulated by motor feedback, (ii) differentially engaged a sparse ensemble reactivation pattern across layer 2/3, which a balanced-state network model reconciled via feedback-induced inhibitory stabilization, and (iii) aligned to regenerative layer 5 apical dendritic Ca^2+^ events. Our results reveal that translaminar spacetime patterns organized by cortical feedback support sparse touch-evoked traveling waves.

## Teaser

Translaminar spacetime patterns organized by feedback-dependent cortical circuits support sparse traveling waves.

## INTRODUCTION

Traveling waves are a fundamental and widespread feature of neuronal activity in mammalian brains (*1*–*9*). These patterns are an emergent property of recurrently connected systems with local recurrent connections (*6*, *10*, *11*) and are believed to play a crucial role in modulating the excitability patterns of cells through precise phase delays, thus coordinating local and global neural activity (*4*). Notably, both evoked and spontaneous traveling waves have been implicated in various cognitive functions such as sensory processing (*1*, *10*, *12*), motor control (*13*), attention, and working memory (*3*). Recent evidence suggests that spontaneous cortical activity is organized in the form of traveling waves, shapes stimulus-evoked responses, and predicts behavioral output (*5*). In each case, the circuit patterns accompanying and supporting traveling waves remain unclear (*7*, *14*), an aspect we address in this study.

There are now two main theories for the neural code underlying sensory-evoked cortical computations: the temporal code and the rate code (*15*). Both of these theories, however, leave open the precise definition of the “window” or “interval of time” over which behaviorally relevant sensory stimuli are encoded in the cortex. By structuring neural activity in single cortical regions across space and time, traveling waves may serve as a substrate to help bind and integrate information over the biologically important periods when sensory-driven computations occur, leading to a spacetime code. Specifically, this spacetime code refers to a situation in which the spike rates and times of a recurrent neural population at any given moment represent sensory-evoked activity that occurred over an interval of time, enabled by waves (*7*, *8*).

How do neural circuits support such waves? Computational evidence suggests that axonal fiber delays due to long-range axons traversing superficial layers in the cortex promote traveling waves (*4*). However, how these traveling waves shape translaminar circuit dynamics and, in turn, how circuit patterns support their propagation remains unknown. Unraveling the link between sensory-evoked traveling wave dynamics and local microcircuit activity is crucial in comprehending how distributed neural computations encode sensory features and shape cognition.

A well-established model for studying sensory processing and related cognitive functions uses the neural response to whisker touch in mice (*12*, *16*). This system is well suited to unmask the neural circuits underlying goal-directed behaviors. Previous studies across the primary whisker somatosensory cortex (wS1) in mice have shown with voltage dye imaging that whisker touch elicits a radially propagating pattern of neural activity that spreads out from the point of maximal input in the cortex (*10*, *12*). Whether this barrel cortex activity is a stationary bump of spreading activity or organized as a wave with precise phase delays has never been experimentally tracked. A single-whisker deflection evokes a sensory response, driven by direct thalamic input, that initiates across multiple layers at its somatotopically aligned barrel in wS1 (*17*). This response rapidly spreads across adjacent barrels (*18*), the secondary whisker somatosensory cortex (wS2), and the whisker motor cortex (wMC) (*12*), including primary and secondary areas. Thus, the whisker barrel represents a powerful system to tease apart the role of short- and long-range connectivity and translaminar circuitry to potential traveling wave spread. On the scale of single cortical regions, experimental evidence suggests that evoked waves allow neural circuits to flexibly store and use information from the recent past, a feature critical to integrating higher-level stimulus features distributed more broadly across space and time (*7*). However, the precise pattern of circuits underlying and supporting touch-evoked wave activity over hundreds of milliseconds remains unknown.

On the basis of data from the barrel cortex, computational analysis suggests that a sparse network of strongly connected excitatory neurons in cortical layer 2/3 (L2/3) drives the emergence of sensory-evoked cortical waves (*10*, *12*, *19*). This model implies that only a small subset of L2/3 neurons is necessary for the propagation of these waves, despite the spatially intermixed response of this subpopulation. Other studies have furthered this model and suggested that these waves propagate through topographically organized, recurrent functional connections in horizontal unmyelinated axons (*4*). The sparse wave here is defined as a network in which wave propagation is supported by just a few local cells (~10%) that spike as the wave passes through (*10*). Although network sparsity in L2/3 is considered a hallmark of traveling waves, observing this “sparse wave” phenomenon directly using conventional recording technologies has been challenging. To do so, one must record superficial traveling waves while simultaneously mapping L2/3 cellular dynamics with a high spatial (cellular and subcellular) resolution.

Previous experiments on traveling waves relied on voltage dye imaging (*14*, *20*), but this technique is now limited to the superficial cortical layers and offers insufficient spatial resolution. While two-photon calcium imaging allows cellular-scale imaging, it cannot capture the temporal dynamics of the wave. Some studies have used Utah arrays (*5*), macroscale electrocorticography (ECoG) arrays (*21*), or microscale ECoG (μECoG) grids (*22*, *23*) to capture traveling waves but have not integrated such recordings with high-resolution functional imaging, limiting the ability to identify the sparse cortical ensembles and translaminar circuits underlying wave structure. In this study, we overcome this challenge in the mouse barrel cortex with a custom fabricated multimodal platform that provides high spatiotemporal resolution wave recordings and cellular-scale two-photon imaging.

We designed, fabricated, and customized flexible and transparent polymer-based NeuroGrids (*22*, *24*) that allow recording traveling waves with high spatial resolution across multiple barrels. By combining this transparent μECoG array with simultaneous two-photon imaging, high-density silicon probe recordings, optogenetics, and pharmacology, we elucidated the translaminar cellular patterns supporting the traveling wave. We directly observed changes in L2/3 sparsity during different traveling wave dynamics. We identified a late, sensory-evoked traveling wave that requires wMC (*25*) and coincides with calcium plateaus in the apical dendrite of layer 5 (L5) neurons (*26*–*28*). We reconcile this motor cortical effect through a balanced-state spiking network model that incorporates realistic neuronal densities, connection probabilities, axonal delays, and synaptic conductances to achieve an inhibitory stabilized network. Together, this study establishes a link between motor cortical feedback, L2/3 network sparsity, and the activation of L5 apical dendrites as key elements underlying stimulus-evoked traveling waves.

## RESULTS

### NeuroGrids map traveling wave dynamics during two-photon imaging

**Fig. 1.**
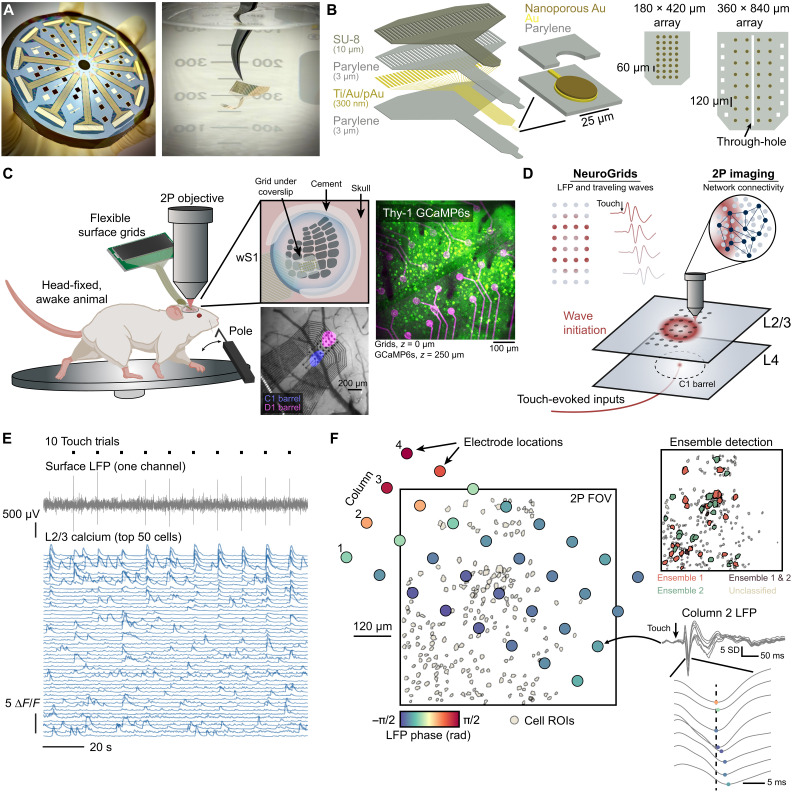
Flexible, transparent grids for simultaneous traveling wave detection and two-photon imaging. (**A**) Left: 4″ Silicon wafer with 12 surface grids fabricated simultaneously. Right: Grid held in water to demonstrate flexibility. (**B**) Left: Cross-sectional stack of the fabricated surface grids. Right: To-scale schematics of the surface grid designs used in this work. (**C**) Schematic depicting simultaneous surface electrophysiology and two-photon imaging. Awake, head-fixed mice run on a wheel and receive either passive or active whisker touch. Surface grids are acutely implanted over wS1. Top inset shows that we place surface grids onto the brain and seal the cranial window with a glass coverslip. The bottom inset is an image of grids on the brain overlaid with the results of intrinsic-optical imaging used to locate the C1 and D1 barrels. Right inset: Validation of the transparent nature of two-photon imaging through the grids. (**D**) Schematic representing different recording schemes. Whisker touch evokes traveling waves in the upper cortical layers that are mapped with NeuroGrids. Concurrently, we also capture cellular-scale network activity with two-photon imaging. (**E**) Example data showing 10 consecutive passive whisker touch trials, LFP recordings from one channel on the grid array, and calcium traces from 50 select cells in the FOV. (**F**) Left: To-scale schematic showing the exact NeuroGrid locations overlaid with the two-photon FOV and detected ROIs 200 μm below the cortical surface. Electrode colormap depicts the average phase of the LFP in a short 10-ms window following touch. Right, top: Same FOV but the cell ROIs are color-coded to match identified ensembles detected with functional connectivity analysis (see Materials and Methods). Ensemble ROIs are increased in size by 200% for clarity. Right, bottom: Representative example showing the average LFP across column two of the electrode array depicting a progressive traveling wave.

We used scalable semiconductor manufacturing to create flexible and semitransparent NeuroGrids (Fig. 1, A and B; see Materials and Methods) (*22*, *23*, *29*). These parylene-based (*30*) μECoG interfaces were patterned with an array of cellular-scale recording sites (25 μm in diameter) coated with a specialized layer of nanoporous gold (*31*, *32*) for low-impedance recording (Fig. 1, B and C, and fig. S1, A to C; see Materials and Methods). Although the NeuroGrids can be customized in several ways such as the length, size, electrode density, and through-hole access, we designed them specifically for high-resolution recordings across two to four barrels in mouse barrel cortex.

The transparency of our NeuroGrids allowed us to combine surface recordings with simultaneous multiphoton imaging (Fig. 1, C to F). After locating the barrel target with intrinsic optical imaging (*33*), we placed NeuroGrids on the cortical surface and sealed across the cranial window with a conventional coverslip (Fig. 1C; see Materials and Methods). This process along with the porous properties of our electrodes allowed us to perform L2/3 two-photon calcium imaging directly through the grids in awake, head-fixed mice (Fig. 1, D and E) with minimal impacts on imaging quality (*34*). Noise artifacts from laser scanning were minimal when imaging GCaMP6s and were easily filtered out with a recursive notch filter (fig. S1, D and E) (*35*). While alternative techniques can be used to further reduce noise artifacts, such as using red-shifted GCaMP sensors or enhancing the electrode transparency with graphene (*29*, *36*), nanomesh arrays (*37*), or other novel materials (*38*–*40*), our recordings revealed that nanoporous gold was sufficient for near artifact-free imaging while allowing for dense patterning. This platform allowed us to record from hundreds of L2/3 cells in the field of view (FOV) with clearly detectable touch-evoked local field potential (LFP) (Fig. 1E and fig. S1, F and G).

Whisker touch (see Materials and Methods) evoked strong LFP and calcium transients throughout the barrel and surrounding areas (Fig. 1, E and F). The LFP was nonstationary and showed clear latencies across the NeuroGrid, indicative of traveling waves (Fig. 1F). By recording simultaneous LFP and cellular calcium in dozens of trials, we could construct maps of LFP latency across the grid in combination with maps of consistent L2/3 network ensembles that arose throughout the recordings (Fig. 1F; see Materials and Methods). This powerful multimodal platform allowed us to dissect the underlying circuit patterns during cortical traveling waves, described below under passive and active touch.

### Passive touch evokes early and late waves in barrel cortex

**Fig. 2.**
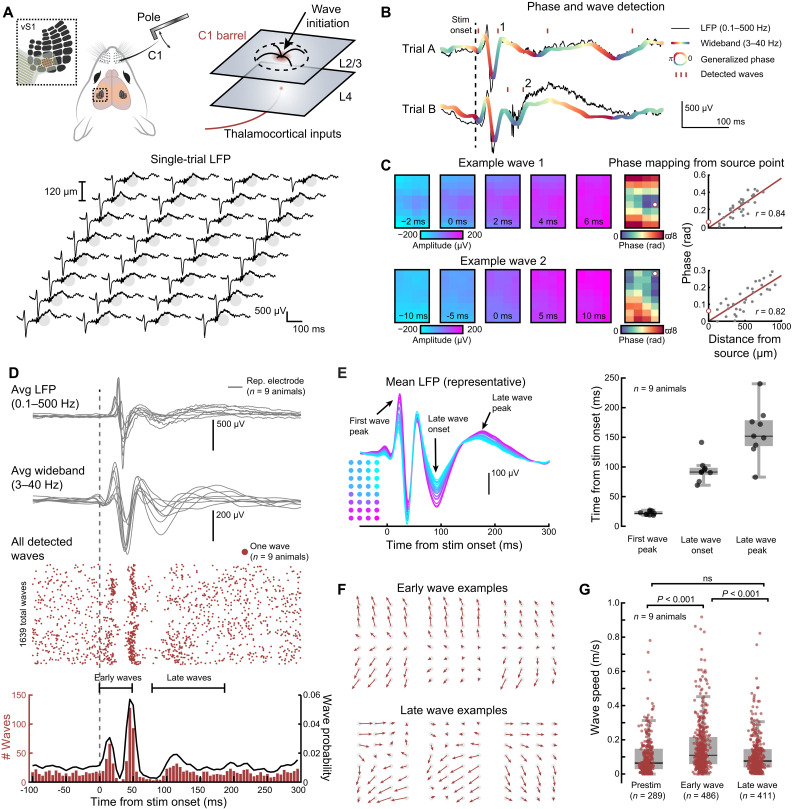
Whisker touch evokes early and late traveling waves across the barrel cortex. (**A**) Top: Schematic of the experimental setup. Bottom: Evoked LFP across the NeuroGrid from a single touch stimulation. (**B**) Detected traveling waves (two touch trials) using GP. LFP is from a representative channel on the NeuroGrid. The gray line indicates the full LFP spectrum (0.1 to 500 Hz). The thick color line is the wideband LFP signal, filtered from 3 to 40 Hz for traveling wave detection and analysis. Colormap is the calculated GP of the wideband signal. Red dashes indicate LFP oscillations that meet the criteria for classification as a traveling wave [see Materials and Methods and fig. S2 (A to C)]. (**C**) Left: Example waves 1 and 2 indicated in (B) traveling across the NeuroGrid. Right: Computed phase map and wave source point (white dot; see Materials and Methods). These act as a snapshot of wave propagation. For traveling waves, the phase across the grid strongly correlates with distance from the source point. (**D**) Average LFP, wideband LFP, and detected traveling waves around the stimulus period for all animals following whisker touch. Only the LFP from one representative channel is shown for each animal. LFP is aligned to the onset of pole motion (*n* = 9 animals, 1639 total detected waves across 607 trials). (**E**) Left: Mean grid LFP (3 to 40 Hz) across all trials for a representative animal with distinct features noted. Color mapping indicates grid location. Right: Quantified times for each feature in the average LFP signal using a representative electrode (*n* = 9 animals). (**F**) Vector plots showing wave propagation in a representative animal. (**G**) Wave speed during the prestimulus, early-evoked, and late-evoked periods (*n* = 9 animals, 289 prestimulus waves, 486 early waves, 411 late waves; *P* < 0.001 Kruskal-Wallis with a post hoc Dunn-Sidak test). ns, not significant.

We first teased apart touch-evoked traveling wave dynamics from an electrophysiological perspective using NeuroGrids (Fig. 2A). In awake animals, passive C1 whisker deflection (see Materials and Methods) drove clear patterns of wave-like of activity across the principal barrel and surrounding columns (Fig. 2A, bottom). Here, passive touch was presented in the absence of reward to avoid confounding effects of anticipation (*41*) and history-based reward accumulation (*42*) on wave dynamics, which can affect cortical responses in a history-dependent manner (*7*, *43*). Averaging LFP across trials can distort and even obscure traveling wave activity (*7*). Therefore, to detect and quantify these waves on a single-trial basis, we used an approach for wave detection called “generalized phase” (GP; Fig. 2B and fig. S2, A to F; see Materials and Methods) (*5*). Like conventional analyses that use a Hilbert transform to attain the analytic signal and instantaneous phase, the GP allows obtaining the instantaneous phase of a wideband signal (3 to 40 Hz; see Materials and Methods) by addressing technical limitations in this signal processing framework. This approach is advantageous, as it can quantify phase (which can, in turn, be used to detect traveling waves) without requiring the assumption that the LFP is narrowband (and, in turn, potentially introducing filtering artifacts). When applied to NeuroGrid recordings, our GP pipeline estimated the instantaneous phase of the wideband LFP and detected both spontaneous and touch-evoked waves (Fig. 2C; see Materials and Methods). Our classification of waves was rigorous and required a consistent pattern of phase delays, or “phase mapping,” from the wave origin point (Fig. 2C and fig. S2, A to C; see Materials and Methods). Across all animals (*n* = 9), we observed spontaneous activity, robustly stereotyped early waves within 50 ms of touch, and a burst of delayed waves (Fig. 2D). These early and delayed waves were present in approximately 60% of trials (fig. S3, A to C). Notably, within the first ~100-ms posttouch, we observed nested epochs with periods comprising waves and no waves (Fig. 2D and fig. S4A). We determined that these epochs to signify traveling waves (i.e., precise phase delays) are intermingled with periods of desynchronized (i.e., unorganized phase) LFPs across the grids, respectively (fig. S4, B and C). Although after ~100 ms, we observed a wave pattern emerges across the surface with a distinct spatiotemporal structure. This delayed potential initiated ~100 ms after touch and peaked ~150 to 200 ms after touch (Fig. 2E). We termed these delayed dynamics “late waves,” and the patterns were distinctly different from the early components in directionality (Fig. 2F). However, we did not probe this aspect further as one would need a larger, high-density grid. Our results, however, do highlight that changes in such patterns could be due to changes in origin which is an active area of investigation.

It is critical to note that this late wave persisted much after the stimulus presentation, suggesting reverberatory effects. We also tested the possibility that changes in locomotion could be attributed to the wave potentials rather than being exclusively touch-induced. However, our mice were stationary and not locomoting during passive touch trials (fig. S2F). Still, this left open the possibility that, in a subset of trials, whisker touch delivered to quiescent animals initiated locomotion and traveling waves. We analyzed LFP and traveling waves in animals transitioning from quiescence to locomotion without touch and found that running reduces the number of detected waves (fig. S2, D and E). Thus, we conclude that the early and late waves are primarily sensory driven.

We quantified speeds in the prestimulus period, early wave period, and during the late wave and found that the early waves traveled slightly faster in comparison to the late wave (Fig. 2G; *P* < 0.001, *n* = 9 animals, 289 spontaneous waves, 486 early waves, 411 late waves, Kruskal-Wallis with a post hoc Dunn-Sidak test; see also fig. S3A). We also quantified the time from wave onset to wave peak, finding that early waves are significantly shorter in their time course than both spontaneous and late waves (fig. S3, A and B; *P* < 0.001, *n* = 9 animals, 289 spontaneous waves, 486 early waves, 411 late waves, Kruskal-Wallis with a post hoc Dunn-Sidak test). These unique properties, in addition to recent works alluding to the role of inhibition in reducing traveling wave speed (*44*) and synchronizing spike timing (*45*), led us to hypothesize that the late wave dynamic could be behaviorally relevant and associated with the spatiotemporal synchronization of barrel cortex circuits.

### Rewarding action in a goal-directed processing task modulates wave dynamics

**Fig. 3.**
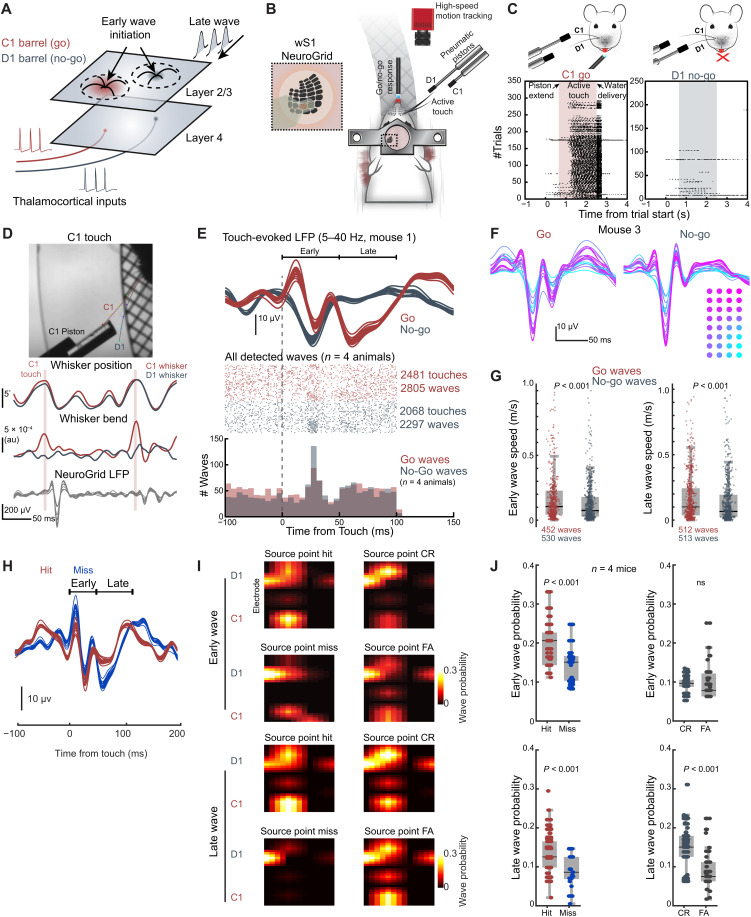
Rewarding action in a go–no-go paradigm modulates early and late traveling wave dynamics during active touch. (**A**) Schematic depicting traveling wave initiation and late wave reverberation in two adjacent barrels. (**B**) Left: Schematic of our two-whisker active touch paradigm. (**C**) Lick raster plot for a representative animal during go and no-go trials. The shaded area indicates when each piston is extended into the whisking field. Black dots are single licks. (**D**) Top micrograph shows a DeepLabCut (DLC) pose estimate of the whiskers during C1 touch. Plots show whisker position (top trace), whisker bending (middle trace), and the touch-evoked LFP (bottom trace). (**E**) Traveling wave detection during C1 and D1 touch. Top: Mean touch-evoked LFP (5 to 40 Hz) for all grid electrodes for a representative animal. Red are go trials, and gray are no-go trials. Middle: Raster plot detected traveling waves across animals during go and no-go trials. Bottom: Histogram of detected waves across animals during go and no-go trials (*n* = 4 animals, 2481 go touches, 2805 go waves, 2068 no-go touches, and 2297 no-go waves). (**F**) Mean touch-evoked LFP for all grid electrodes for a representative animal. LFP is color-coded to the electrode location. (**G**) Early and late wave speed between go and no-go trials (*n* = 4 animals, 452/512 early/late go waves, 530/513 early/late no-go waves, *P* < 0.001, rank sum Wilcoxon test). (**H**) Surface LFP response during hit-and-miss response across the grid for the go whisker (C1). (**I**) Probability of wave source points for a representative grid covering both barrels [Go- Hits; No Go- Correct Reject (CR); FA, False alarms]. See approximate grid orientation in (B). (**J**) Top: Early traveling wave probability for each task outcome (*n* = 4 animals, 2481 go touches and 2068 no-go touches). Bottom: Late traveling wave probability for each task outcome [*n* = 4 animals, 452 go (hit) waves, 530 no-go (CR) waves, rank sum Wilcoxon test]. au, arbitrary units.

Our results with passive touch stimulation led us to probe whether traveling waves across the barrel cortex can reflect behaviorally relevant information under active paradigms where volitional movement of whiskers against an object occurs. We hypothesized that reward-based reinforcement would modulate wave properties toward dynamics that enhance the saliency of a reward stimulus under active touch. To test this, we developed a two-whisker, active touch discrimination task (Fig. 3, A to C, and fig. S5A; see Materials and Methods). In head-fixed animals, we targeted the C1 and D1 whiskers separately with a system of pneumatic pistons (*46*) that animals were free to actively whisk against for 1.3 s (Fig. 3, B to D, and fig. S5A; see Materials and Methods). We trained animals in an operant task to lick for a water reward during C1 touch (go stimulus) and withhold licks during D1 touch (no-go stimulus; Fig. 3C and fig. S5A). In this scenario, go and no-go trials should evoke waves of activity, including actively engaging higher-order feedback in the C1 and D1 barrels, allowing us to test for goal-directed changes to traveling waves (Fig. 3A). This is further strengthened by the growing evidence that higher-order wMC feedback plays a role in shaping behavior (*47*) and is fundamental to suppressing distractor stimuli in the barrel cortex (*48*)

After training animals to expertly discriminate (fig. S5B), we performed NeuroGrid surface recordings and high-speed whisker imaging as animals undertook the behavioral task. We then used whisker tracking with DeepLabCut (DLC) (*49*) to detect the precise C1 and D1 touch times during go and no-go trials (Fig. 3D; see Materials and Methods). As with passive touch recordings, we used our GP pipeline to detect spontaneous and touch-evoked traveling waves in the wideband filtered LFP throughout the experiment. We restricted our analysis to consecutive touches with an interval greater than 100 ms to remove the confounding factor of multiple touches happening in rapid succession, which we believed would interfere with late wave detection. We found that only the first touch resulted in an appreciable surface potential swing, while rapid consecutive touches created reduced depolarizations for both go and no-go trials (fig. S6).

Mirroring our results with passive touch, across all animals we detected both early (<50 ms after touch) and late (50 to 100 ms after touch) touch-evoked traveling waves in both go and no-go trials (Fig. 3E; *n* = 4 animals, 2481 C1 touches, 2068 D1 touches, 2805 go waves, 2297 no-go waves). We confirmed that these waves results are touch related by analyzing waves during free whisking in the absence of touch and did not observe a strong correlation between LFP potentials or traveling wave onset times and whisking phase (fig. S5, C and D; *n* = 5 animals, 5507 total whisking cycles, 537 total detected waves). We also found that touch-evoked waves exhibited significantly larger LFP amplitudes (fig. S5D; *n* = 5 animals, 537 whisking-only waves, 1275 early waves, 963 late waves). We did observe that the late wave analysis window (Fig. 3E; 50 to 100 ms) was at odds with the results in Fig. 2D (passive touch), where 50 to 100 ms is the period with the least number of waves detected before persistent wave activity was observed. Given the trough to peak in the surface potential signaled the onset of waves, we surmised that the LFP and wave structure during active touch are more compressed as the trough to peak occurs earlier, possibly due to differences in brain state and local circuit dynamics.

How does goal-directed behavior shape touch-evoked phase dynamics within the barrel? We quantified specific properties of phase across the NeuroGrid during go and no-go trials to determine the effects of sensory associations on traveling waves. We discovered a notable difference in wave speed across the NeuroGrid, specifically faster early and late waves during go trials compared to no-go trials (Fig. 3G; *n* = 4 animals, *P* < 0.001, rank sum Wilcoxon test). Faster waves suggest increased phase alignment and higher synchronization across the barrel during go trials. We also noted a significant enhancement of the late LFP for the go stimulus (fig. S5, E and F) (*n* = 4 animals, *P* < 0.001, Wilcoxon signed-rank test). These results indicate that rewarding action drives distinct wave properties, likely influencing synchronization across the translaminar axis, and that the late wave may be a distinct mechanism for more accurate sensory perception, particularly because whisker movement was largely stereotyped across both the go and no-go trials (fig. S5, G to J).

Upon further analysis, and notably, we found that traveling wave source points were distinctly localized to barrels, which encoded hits, misses, and correct rejects (Fig. 3, H and I). A clear difference in the wave structure (timing, amplitude) and probability was observed for the hits versus misses (Fig. 3H), most noticeable during the late wave period across the principal whisker column (Fig. 3I, bottom), an effect one would not observe by just measuring LFP alone. Moreover, the timescales of the wave and animal behavior suggest that the late wave may be a critical circuit feature for ensuring the correct initiation or withholding of a lick. Our data show that waves are most strongly evoked by touch, not by reward anticipation or licking (fig. S7A). The sensory-evoked waves occured over a ~200-ms window, and anticipatory licking ensued approximately 500 ms after the first touch and lasted ~1 s until reward delivery (Fig. 3C and fig. S7A). We also observed a slight sharpening of lick onset when the first wave occurred within 300 ms after the first touch (fig. S7, B to D). This timeline suggests that the late wave dynamic may not only be a distinct signature of salient stimuli but also an important feature needed for optimal signal routing to ensure an impending lick or an intent to withhold response. Our recordings reveal that traveling wave structure is modulated by sensory associations. Overall, the wave property and not just the amplitude of the LFP—most notably during the late period—was strongly predictive of future task outcome (Fig. 3J). These experiments demonstrate that early and delayed sensory-evoked waves are apparent during active touch and carry context-dependent information associated with impending behavior.

### Touch-evoked waves across frequency bands reveal translaminar circuit contributions

Next, we focused on dissecting the underlying circuit dynamics that support a wave structure. We used single-whisker passive touch due to its tractability and simplicity for in-depth circuit interrogation. We analyzed specific frequency bands that are known to be associated with signal routing across specific cortical layers (*50*). How does each frequency band support touch-evoked traveling waves observed on the NeuroGrid?

**Fig. 4.**
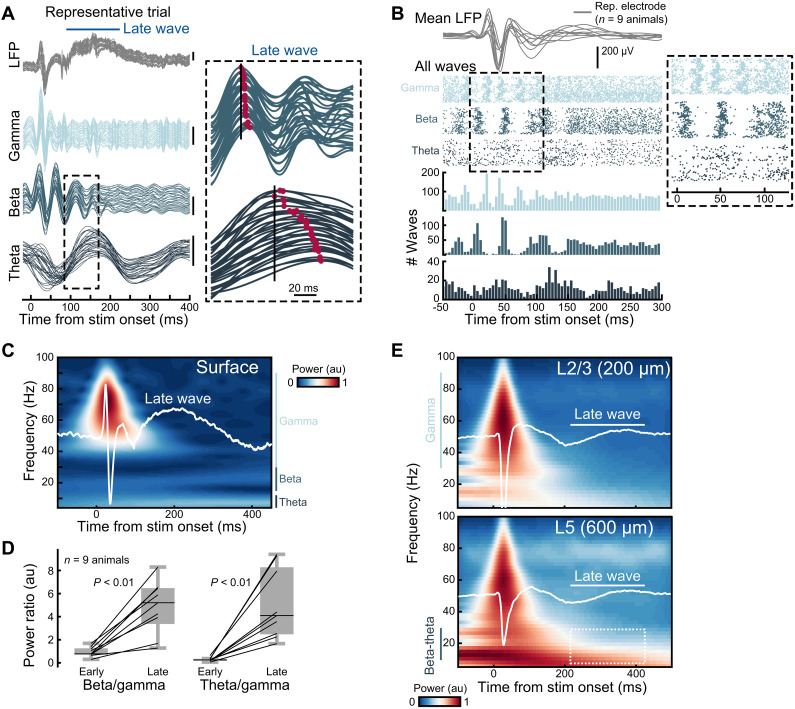
The beta and theta bands contribute to the late wave. (**A**) Single-trial LFP and the corresponding gamma (30 to 90 Hz), beta (15 to 30 Hz), and theta (4 to 12 Hz) frequency bands. The inset shows the beta and theta bands during the onset of the late wave. Red denotes the local maxima and shows wave latencies across the grid. (**B**) Detected traveling waves in the gamma, beta, and theta bands for all animals (*n* = 9 animals). Top traces show each animal’s mean touch-evoked LFP on the NeuroGrid on a representative electrode. The raster plot shows the onset times for all detected waves. Inset shows a zoom of the first 100 ms. The histogram shows the combined wave times across animals (*n* = 9 animals, 14,598 gamma waves, 7100 beta waves, and 2529 theta waves). (**C**) Mean touch-evoked LFP on the NeuroGrid for a representative animal overlaid on the signal spectrogram. (**D**) Beta/gamma and theta/gamma power ratios across animals in the early (0 to 50 ms) and late (100 to 250 ms) windows following whisker touch (*n* = 9 animals, *P* < 0.01 signed-rank Wilcoxon test). (**E**) L2/3 and L5 touch-evoked spectrogram for a representative animal from silicon probe recordings (*n* = 3; see fig. S8). The late wave appears ~200 ms after touch and most clearly correlates with delayed beta-theta coupling in L5.

To reveal this, we reanalyzed traveling waves during the early and late periods but with an emphasis on the narrowband frequencies involved (Fig. 4 and fig. S8A). Decomposing the full LFP spectrum into the gamma (30 to 90 Hz), beta (15 to 30 Hz), and theta (4 to 12 Hz) bands (see Materials and Methods) exposed a clear frequency dependence of the early and late waves, with an emphasis of the beta and theta bands on the late period (Fig. 4A). When we detected traveling waves with our GP pipeline, we observed an intricate assembly of touch-evoked wave events across frequency bands (Fig. 4B; *n* = 9 animals, 14,598 gamma waves, 7100 beta waves, 2529 theta waves). Here, we observed that epochs of waves in the gamma band appeared interleaved with epochs of beta waves (Fig. 4B, inset). Here, we observed that waves in the gamma band appeared to be nested within periods of beta waves. As with the wideband-filtered waves described in Fig. 2, the median traveling wave speed of all detected narrowband waves falls within the reported range for unmyelinated axons (<1 m/s), with a progressive slowing in wave speeds in lower-frequency bands (fig. S8A; *n* = 9 animals, 14,598 gamma waves, 7100 beta waves, 2529 theta waves).

These interesting frequency-specific properties hint that the underlying circuits supporting sensory-evoked waves at the cortical surface are translaminar. Most conspicuously, with NeuroGrid recordings, we found that the early-evoked waves (0 to 50 ms) are dominated by gamma-beta frequency bands, while the late wave (100 to 300 ms) is dominated by lower frequencies in the beta-theta range (Fig. 4, C and D; *n* = 9 animals, *P* < 0.01, signed-rank Wilcoxon test). Previous studies in nonhuman primates have found a cortex-wide motif in which supra-granular circuits (L2/3) are dominated by the gamma band and infragranular layers (L5/6) largely by beta and theta frequencies (*50*–*52*). This translaminar motif is also present in mouse visual cortex during visual stimulation, albeit to a less prominent extent (*53*). We performed similar frequency analyses following silicon-probe recordings of touch-evoked LFP in mouse barrel cortex, revealing that deep cortical layers exhibit low frequencies in the beta-theta range that are more prominent than the gamma band (fig. S8B; *n* = 3 animals). These experiments also revealed another important layer-specific motif. The late wave period (100 to 300 ms after touch) showed a marked increase in beta-theta power, but this low-frequency increase was largely restricted to the infragranular layers (Fig. 4E). These combined observations suggest that the waves observed at the cortical surface involve an intricate translaminar coupling wherein infragranular circuits are important for the supragranular wave. Specifically, they point to distinct contributions across superficial and deep layer neurons, suggesting that a precise spacetime code supports traveling wave dynamics and spread.

### L2/3 sparsity hallmarks traveling wave dynamics

**Fig. 5.**
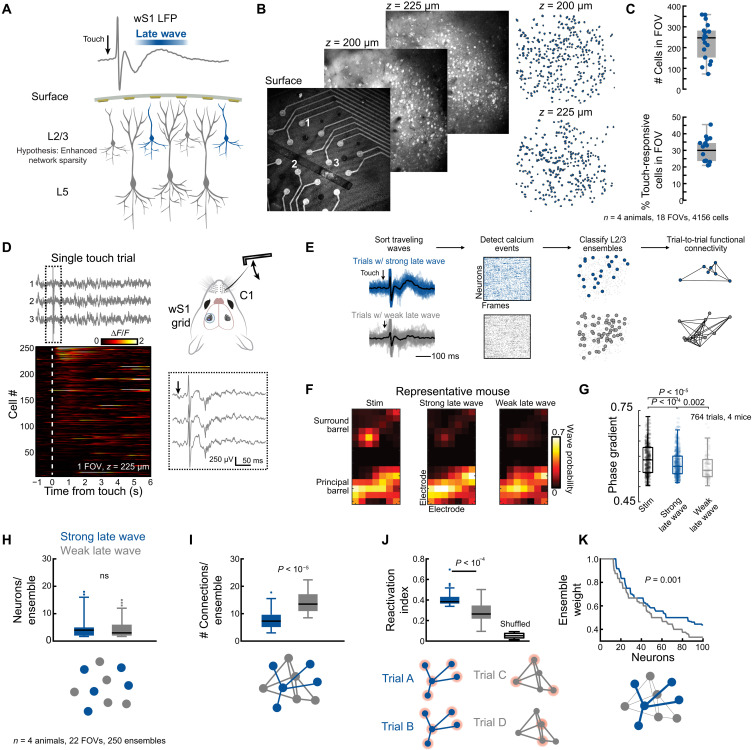
Enhanced L2/3 sparsity supports late wave dynamics. (**A**) Schematic of a sparse network theory in L2/3 that supports the late wave. (**B**) Representative wS1 two-photon imaging through the surface grid. Left: Images 200 and 225 μm below the grids. Right: Cell ROIs at each depth. (**C**) The number of cells in each FOV across animals and the percent of touch-responsive cells in each FOV (see Materials and Methods; *n* = 4 animals, 18 total FOVs, 4156 total cell ROIs). (**D**) Representative C1 touch trial during simultaneous surface electrophysiology and 2P imaging (*z* = 225 μm). The traces correspond to the labeled electrodes in (B). The inset shows the traces immediately following touch. (**E**) 2P data analysis pipeline for detecting L2/3 neuronal ensembles and quantifying functional connectivity on a single-trial basis (see Materials and Methods and fig. S9). (**F**) Traveling wave spatial source points in a representative mouse during stimulation (early) and late response. Note that only the principal whisker is stimulated. (**G**) Differences in wave phase-gradient magnitude across timescales of the principal barrel (rank sum Wilcoxon test, 764 trials, *n* = 4 mice). (**H** to **K**) Quantified results from functional connectivity analysis (*n* = 4 animals, 22 FOVs, 250 total detected ensembles). (H) The total number of cells within each ensemble was unchanged for ensembles associated with the late wave or a weak late wave (rank sum Wilcoxon test). (I) The number of functional connections within the ensembles is higher for weak late wave trials, suggesting that increased sparsity supports the late wave (*P* < 0.001, rank sum Wilcoxon test). (J) The late wave ensembles show a significantly higher reactivation index. Randomly shuffling the calcium events abolishes the reactivation correlations (*P* < 0.001, Kruskal-Wallis with a post hoc Dunn-Sidak test). (K) The late wave ensemble shows consistently higher functional connectivity weights (*P* < 0.001, Kolmogorov-Smirnov test).

We next sought to directly map layer-specific circuit dynamics underlying traveling waves, beginning with L2/3. Network sparsity in L2/3 has been implicated as a critical component underlying traveling wave propagation, albeit computationally (*10*); however, existing experimental methods lack the multimodal capability necessary to directly observe this phenomenon. Here, we leveraged the transparency of the NeuroGrid array to combine our traveling-wave recordings with simultaneous two-photon imaging of L2/3 dynamics (Figs. 1, C and F, and 5; see Materials and Methods). Specifically, we hypothesized that different traveling wave dynamics—namely, trials with and without the prominent late wave—would be supported by different L2/3 circuits and possibly changes in network sparsity (Fig. 5A). The presence and absence of a late wave occurred randomly, interleaved within trials in which there were appreciable late wave dynamics (52 ± 27% of all trials, 67 ± 21% of trials with a clearly detectable touch-evoked LFP, mean ± SD, *n* = 13 animals). Waves were not linked to spontaneous whisking (fig. S5C) or evoked by behavioral state transitions (fig. S2, D and E). Furthermore, since mice were stationary during passive whisker touch (fig. S2F), our results are not confounded by state changes related to locomotion. We thus hypothesize that the absence of late waves was most likely due to periodic brain state changes occurring on a trial-to-trial basis. Through the NeuroGrids, we consistently observed a strong touch-evoked LFP while imaging imaged hundreds of L2/3 cells at depths of 150 to 250 μm with a minimal impact on the number of cells in the FOV, calcium signal-to-noise ratio, and number of touch-responsive L2/3 cells (Fig. 5, B and C, and fig. S1).

To approach the complex task of comparing the slow, cell-specific calcium dynamics of two-photon imaging with the high-speed LFP recordings of the NeuroGrid, we developed an analysis pipeline focused on a trial-by-trial analyses of neuronal ensembles (Fig. 5, D and E, and figs. S9, A to D, and S10; see Materials and Methods) (*54*, *55*). This approach overcomes the limited value of a frame-by-frame comparison of slow calcium dynamics to electrophysiology. Specifically, we sorted trials into categories based on the strength of the late wave (Fig. 5E), which generated different wave LFP profiles—trials with a strong late wave and trials with a weak late wave. After making this distinction, we noted that the early and late responses of all waves were strongly linked to the principal whisker barrel (Fig. 5F); we also noted that although activity was most prominent in the principal whisker, a small number of waves were also found in the adjacent barrel. To quantify the behavior of propagating waves during epochs of the trial, we computed the phase-gradient directionality (PGD) across trials (see Materials and Methods). PGD quantifies the degree of alignment of the phase gradients across the array, and a high PGD indicates a well-defined propagation direction (*13*). Specifically, we found that weak late waves exhibited a reduced PGD when compared to either the strong late waves and early waves (Fig. 5G; *P* < 0.002 rank sum Wilcoxon test). Therefore, the amplitude of the late potential is indicative of the magnitude of the wave phase gradient. How are the L2/3 cellular populations altered during these changes in wave spatial dynamics? For each LFP category, we gathered all calcium events for cells in the FOV beginning 500 ms before touch and ending 4 s after touch, used a vector-based covariance analysis to cluster cells into neuronal ensembles, and ran a trial-by-trial functional connectivity analysis on the detected ensembles (Fig. 5E and fig. S9, A to D; see Methods). These analyses allowed for the quantification of connectivity patterns in prominent ensembles that arose in trials with and without the late wave, thus enabling us to gauge L2/3 sparsity based on traveling wave spatial properties (fig. S10).

Across four animals and 22 FOVs at varying L2/3 depths, we detected 250 total neuronal ensembles. The average size of these ensembles was small (6 ± 3 neurons, 7 ± 3 neurons across 22 separate fields of views for trials containing a strong late and weak late wave, respectively) and did not change in trials with and without a strong late wave (Fig. 5H; *P* > 0.05, rank sum Wilcoxon test). However, the number of functional connections in each ensemble was reduced by nearly 50% in trials with a prominent late wave, indicating enhanced sparsity (Fig. 5I; *P* < 0.0001, rank sum Wilcoxon test). Despite these reduced connections, the late wave ensembles showed a markedly increased reactivation index, a measure of the reactivity of the ensembles on a frame-by-frame basis, compared to chance (Fig. 5J; *P* < 0.0001, Kruskal-Wallis test with a post hoc Dunn-Sidak test). Conversely, ensembles during trials with weak late waves were more variable, reflective of lower functional connectivity resulting in a lower reactivation index (Fig. 5J). These results suggest that an increase in the strength of functional connections in the ensembles was dependent on whether touch trials contained a strong late wave or a weak late wave component. Neuronal ensembles associated with the late wave showed significantly increased connectivity weights, a measure of the strength of individual connections between pairs of neurons across ensembles (Fig. 5K; *P* < 0.001, Kolmogorov-Smirnov test; fig. S10, I to K). These results prove that a “sparse but strong” functional connectivity framework supports traveling waves across L2/3—particularly during waves with heightened spatial phase gradients—and provide the first, to our knowledge, direct experimental demonstration for the sparse wave theory (*7*).

### A recurrent circuit model reconciles feedback control of sparsity

How might the late wave control wS1 sparsity? Our narrowband analysis revealed nested beta-theta wave dynamics upon touch and that extended hundreds of milliseconds posttouch (Fig. 4). Given beta is a hallmark of top-down input and goal-directed processing (*50*, *51*, *56*), we turned our attention to ascertain the effect of feedback inputs on sparsity.

**Fig. 6.**
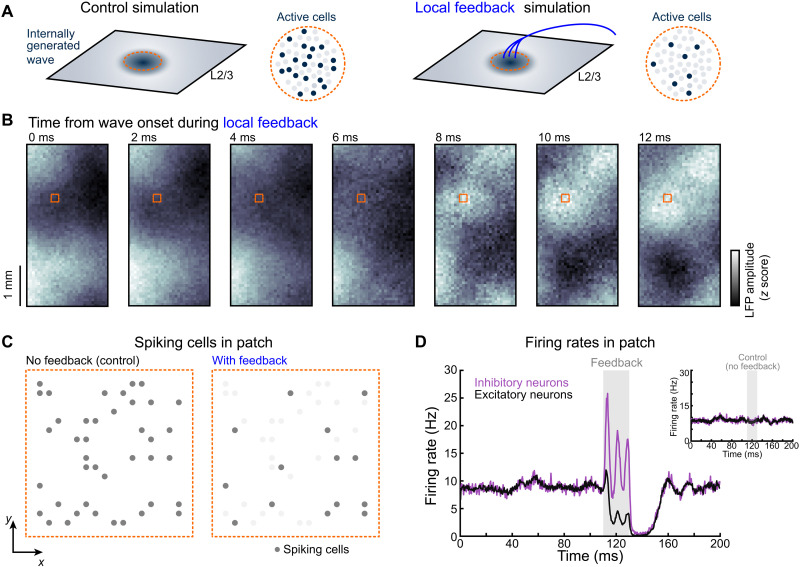
An E-I balanced model reproduces L2/3 sparsity through feedback wave-induced inhibitory stabilization. (**A**) Schematic overviewing the spiking neural network stimulation and results. (**B**) Spontaneous L2/3 traveling wave LFP modeled on a 2 mm-by-4 mm patch of cortex. A *t* = 0 ms, a sensory stimulus and feedback inputs are exerted onto a small cortical patch (dotted square box). Notice how the wave changes as a function of time. (**C**) Zoom on a 100 μm-by-100 μm area of modeled cortex indicated by the dotted square box in (B) to show the responses of single cells during simulations with and without feedback. (**D**) Firing rate in the local network with and without feedback inputs. Feedback inputs during the wave decrease spiking activity in the local population (main), in contrast to the same network without feedback, which shows smaller fluctuations in spike rate (inset).

**Fig. 7.**
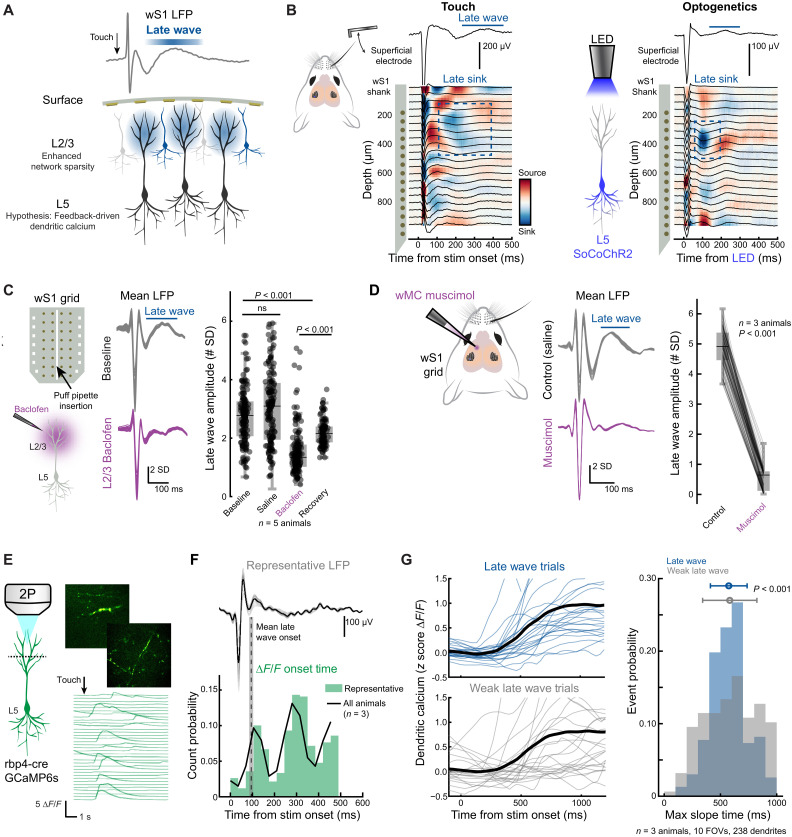
The late wave sharpens L5 dendritic calcium spikes. (**A**) Schematic outlining the hypothesis that L5 dendrites contribute to the late wave. (**B**) Current source density (CSD) analysis and simultaneous NeuroGrid recordings in wS1 during C1 touch (left) and L5 optogenetic stimulation (right) for a representative animal. (**C**) Left: Schematic of the approach with baclofen. Middle: Average LFP across the grid from a representative animal. Baseline recordings (gray) and after baclofen injection (purple). Right: Late wave amplitudes during baseline recordings, a control ACSF injection, baclofen injection, and recovery after wash-out. Each data point is a grid channel [*n* = 5 animals, *P* < 0.0001, Friedman test with a post hoc Dunn-Sidak test; see fig. S13 (A to C) for more results]. (**D**) wS1 NeuroGrid recording during wMC silencing with muscimol abolishes the late wave. Each line indicates a single grid channel (*n* = 3 animals, *P* < 0.001, signed-rank Wilcoxon test). (**E**) Representative touch-evoked Δ*F*/*F* transients from an apical dendrite. (**F**) Simultaneous LFP recordings and L5 apical calcium transient detection. Top: Representative mean surface potential during C1 touch for a representative animal. Bottom: Dendritic calcium onset times across all animals (*n* = 3 animals, 238 total dendrites). (**G**) Left: Mean touch-evoked transients sorted into trials with and without a prominent late wave for a representative animal. Dendrites shown are the top 50th percentile of touch-responsive cells. Right: Histogram of the max dendritic Δ*F*/*F* slope times along for trials with and without a strong late wave (*n* = 3 animals; 238 total dendrites and 232 dendrites showed responses during late wave trials, and 157 dendrites showed responses during trials with a weak late wave). The average transient time between distributions is the same (*P* = 0.57). The SD of the late wave distribution is smaller (*P* < 0.001, rank sum Wilcoxon test following bootstrapping).

We hypothesized that feedback inputs could further enhance sparsity in L2/3 populations by primarily targeting inhibitory neurons. To test this hypothesis, we used a spiking network model, reminiscent of L2/3, operating in a state of balanced excitation and inhibition (Fig. 6A). Synapses in the network were conductance based. These synaptic interactions allow the modeled networks to produce self-sustained, low-rate, asynchronous activity consistent with the statistics of the spontaneous L2/3 background state in vivo. These synaptic interactions, which are considered the driving force of excitatory and inhibitory conductance, allow networks to produce robust self-sustained activity, in which the network can self-generate low-rate asynchronous-irregular activity consistent with the statistics of the spontaneous background state in vivo (*57*, *58*). At scales matching the biological networks in our experimental recordings, these self-sustained states can occur with synaptic interactions in the range of 0.15-mV excitatory postsynaptic potential from rest, matching the scale of single inputs in vivo (*58*). This means that neurons in the model experience synaptic inputs and conductance states approximating what occurs in awake, behaving animals (*59*–*61*). The addition of local connectivity, where the likelihood of connection decreases as a Gaussian with the distance between two neurons, and distance-dependent time delays that simulate the conduction of spikes along the unmyelinated horizontal fibers connecting neurons across L2/3 results in the organization of self-sustained activity into waves that systematically travel across the network (Fig. 6B) (*2*, *4*). Waves in this network excite neurons as they pass over a local area in the network (gray dots, Fig. 6C). We model the feedback experimentally observed from wMC as a set of fibers (indicated in blue, Fig. 6A, right) providing input to excitatory and inhibitory populations at a local patch of the model (orange square indicated in Fig. 6B). We then use this network model to test whether feedback could sparsify spiking activity during a wave similar to the ensemble dynamics during the late wave observed in experiments. We find that predominantly inhibitory-targeted feedback occurring as an individual wave passes over a local network can substantially increase the sparsity of traveling waves in the network (Fig. 6C). The feedback connections have a rate and hold systematically for feedback targeting different ratios of inhibitory-to-excitatory neurons (fig. S11B). Through systematic numerical study, we identify the precise point in parameter space where descending feedback projections cause a decrease in spiking in the target population (Fig. 6D). This decrease does not occur in the absence of feedback activity (Fig. 6D, inset), demonstrating that the feedback activity specifically causes a decrease in the spike rate of this local population. Notably, the cessation of the feedback inputs leads to a transient reduction in both the excitatory and inhibitory firing rates, reminiscent of inhibitory stabilization (Fig. 6D) (*62*). Overall, the simulations show that descending feedback projections could further increase the sparsity of these ongoing waves, creating an even more “sparse” wave, in that relatively fewer neurons spike reliably as the wave propagates, a feature we believe is mirrored by the increased ensemble reactivation/stability observed in the experimental data during the strong late wave (Fig. 5). Such an effect could be obtained via top-down wMC feedback in wS1 (*25*). Silencing wMC with muscimol decreased ensemble stability in L2/3 (fig. S11A, *P* < 0.001, *n* = 4 mice), suggesting that wMC feedback to wS1 plays a critical role in sparsifying the L2/3 response. But the question remains, how is the prominent surface potential during the late wave produced if the population level response in L2/3 is sparsified as a function of feedback? We hypothesize that wMC feedback sculpts and modulates the late wave in wS1 by simultaneously controlling L2/3 sparsity (Fig. 5) and gating L5 apical dendritic calcium activity (Fig. 7A), reflected in the cortical surface potential. We examine this effect below.

### Motor feedback and L5 Ca^2+^ spikes modulate the late wave

Our theoretical results (Fig. 6) demonstrated a potential mechanism for how feedback can influence stimulus triggered traveling waves that results in the control of sparsity in L2/3 activity across wS1. If the late wave is wMC feedback modulated, then what is the precise source of this superficial wave in the barrel cortex? The sparsity of L2/3 (<10 cells per ensemble) suggests an alternative origin than somatic components in the supragranular layers. Our narrowband frequency analysis and laminar recording open the possibility of an infragranular contribution to this potential via a prominent beta component (Fig. 4, C to E). Yet, it is unlikely that NeuroGrids capture infragranular somatic activity due to the distance of the cell bodies from the cortical surface (>500 μm) (*63*). However, it could be possible that infragranular dendritic projections to the superficial layers—which exhibit a variety of calcium-driven regenerative spikes—contribute to the activity observed on the NeuroGrid (*28*). Previous reports have suggested that somatic activity cannot fully explain delayed potentials driven by sensory input and is likely dendritic in origin (*64*). In wS1, in addition to having a depolarization effect on L2/3 (*65*), studies have demonstrated the critical requirement of wMC feedback in gating L5 dendritic activity (*26*, *47*). This is further substantiated by the fact that direct wMC inputs to wS1 synapse onto both L5 apical and somatic compartments (*66*), suggesting coincidence detection, while also indirectly targeting L5 activity via the secondary thalamic nuclei (posteromedial thalamic nucleus POm) (*67*). Moreover, this premise of a dendritic origin to surface potentials is also prevalent in human recordings (*68*).

We began testing this hypothesis by locating the anatomical depth of the late wave with silicon depth probes and current source density (CSD) analysis (Fig. 7B and fig. S12, A to C). In line with previous studies (*69*), we found that whisker touch drives not only an array of early sinks and sources consistent with the canonical cortical circuit but also an extensive and delayed L2/3 current sink associated with positive potentials in the superficial layers (Fig. 7B and fig. S12, A to C). In simultaneous NeuroGrid and silicon probe recordings, we found that this delayed L2/3 sink disappeared in trials without the late surface wave, indicating that the late superficial sink is tightly linked to the late wave (fig. S12, B and C; *n* = 4 animals, *P* < 0.001, signed-rank Wilcoxon test). While the late L2/3 sink could be due to somatic spiking, we did not find a significant difference in single-unit activity firing rates in the supragranular layers across trials exhibiting a strong and weak late sink (fig. S12, D to F; *n* = 4 animals, *P* = 0.36, signed-rank Wilcoxon test). These results suggest that superficial excitatory activity cannot fully explain the delayed L2/3 current sink. However, it is possible that L5 somatic activity drives calcium electrogenesis in superficial apical dendrites, which contributes to L2/3 current sinks (*28*). When we optogenetically activated wS1 L5 somatic activity (see Materials and Methods), we recapitulated a delayed supragranular current sink (Fig. 7B). Moreover, in opposition to L2/3, L5 neurons showed an increase in single-unit firing rates during the late period in trials with the late sink (fig. S12, E and F; *n* = 4 animals, *P* < 0.01, signed-rank Wilcoxon test). These experiments strongly implicate L5 apical dendrites in the late wave/current sink. To test their role even more directly, we pharmacologically silenced L5 calcium electrogenesis with the GABA_B_ agonist baclofen injected 250 μm below the NeuroGrid (Fig. 7C and fig. S13, A to E; see Materials and Methods), which has been previously shown to primarily influence dendritic burst spiking (*70*). For a period, this markedly reduced the late wave amplitude before partially recovering after wash-out (Fig. 7C; *n* = 5 animals, *P* < 0.001, Friedman test with a post hoc Dunn-Sidak test) while largely preserving the early wave potential (fig. S13, A to C) and preserving wave properties such as speed and duration (fig. S13, D and E). These experiments support a circuit model in which the late wave is strongly composed of signals originating from L5 apical dendritic Ca^2+^ spikes.

Consistent with this hypothesis of wMC initiated L5 dendritic spikes contributing to the late wave, we were able to abolish the late wave amplitude by pharmacologically silencing wMC with muscimol (Fig. 7D; *n* = 3 animals, *P* < 0.001, signed-rank Wilcoxon test). Furthermore, optogenetic inhibition of wMC with the light-gate chloride channel NpHR3 expressed in excitatory neurons (see Materials and Methods) also reduced the late wave amplitude (fig. S13, F to H; *n* = 3 animals, *P* < 0.001, signed-rank Wilcoxon test). While these experiments suggest that a complex interaction between L2/3 subnetworks and L5 neurons are the origin of the wS1 late wave, they do not explain the large delay of this large amplitude traveling potentials posttouch. This variable delay (~100 to 200 ms; see Fig. 2) suggests that a reverberatory circuit loop may underly this phenomenon (*71*). To test this, we performed optogenetic activation of wMC with channelrhodopsin expressed in excitatory cells which drove a delayed supragranular current sink in wS1 approximately 100 to 200 ms after light stimulation, consistent with the delayed timing of sensory-evoked L5 apical dendritic regenerative currents (fig. S13, I to J; *n* = 3 animals). These experiments demonstrate that the late wave is dependent on wMC feedback, is tightly linked to L5 dendritic calcium spikes, and clearly visible from the surface of the brain.

### The strength of the late wave coincides with L5 output

How does the late wave map to L5 dendritic activity? To directly probe this relationship, we again turned to simultaneous NeuroGrid recordings with two-photon imaging during whisker touch (Fig. 7E; see Materials and Methods). Dendritic calcium transients were readily measurable via transgenic lines (Rbp4-cre) labeled with GCaMP6s, and the signals were visible through the NeuroGrid (Fig. 7E). While the decay time of GCaMP6s is slow, the transient onset time is known to be a close measure of electrophysiology onset and is sensitive to single action potentials under favorable conditions (*72*). Therefore, we compared dendritic Δ*F*/*F* onset times to surface grid recordings and found the dendritic Ca^2+^ transient activation time closely tracked the late wave onset (Fig. 7F; *n* = 3 animals, 238 dendrites). These data also show that L5 dendritic spike onset times can often occur even 300 ms after touch, and we observe a smaller secondary positive surface potential swing on the NeuroGrid (Fig. 7F). These results match well with recent studies in mouse visual cortex that demonstrate that visual stimuli can drive multiple low-frequency waves hundreds of milliseconds following sensory input (*73*).

As with experiments in L2/3, we sorted NeuroGrid trials with simultaneous dendritic imaging into LFP that showed strong and weak late waves (*n* = 3 animals), where we previously found that strong late waves generated enhanced phase spatial profiles (see Fig. 5G). In trials with prominent late waves, dendritic transients were more synchronized upon whisker touch (Fig. 7G, top), in comparison to trials without a prominent late wave (Fig. 7G, bottom). When we quantified the rising phase of dendritic calcium transients across animals, we discovered a significant sharpening of dendritic responses (Fig. 7G, right; *n* = 3 animals, 10 FOVs, 238 dendrites, *P* < 0.001, rank sum Wilcoxon test). Our results show that wMC feedback—possibly by recruiting local inhibitory circuits in sensory cortex—shapes traveling wave structure by sculpting translaminar activity. This result also establishes how traveling waves compose of distinct feedforward and feedback components over different timescales.

## DISCUSSION

In this work, we provide evidence that distinct translaminar circuit patterns with both cellular and subcellular origins support cortical surface traveling wave dynamics across the barrel cortex of mice. We introduced a custom, multimodal, transparent, and flexible NeuroGrid platform and dissected the translaminar circuit components underlying sensory-evoked traveling waves. Our results provide direct evidence that (i) touch-evoked traveling waves across barrel columns compose of distinct feedforward and feedback contributions, (ii) sparse representations in L2/3 pyramidal cells coincides with traveling wave propagation across the cortical surface, and (iii) traveling waves influence cortical output by shaping L5 timing (Fig. 7). These results, in combination with our observation of wave modulation by reward-based associations, provide a link for how cortical waves correlate and carry with them information relevant to sensorimotor behaviors, stimulus representations, and sensory associations.

### Whisker touch–evoked traveling waves can affect cortical coding

The LFP is produced by synaptic inputs, which cause variable fluctuations in spontaneous activity and stimulus-evoked responses. These fluctuations are governed primarily by the local recurrent connectivity of cortical networks, which induces waves traveling across cortical space (*7*). Previous studies have established that these waves occur spontaneously in awake behaving conditions (*4*) and that strong driving input, such as whisker touch, is believed to reduce variability in ongoing dynamics, producing strong correlations in spiking activity. This raises questions about the potential impact of traveling waves on evoked activity. Our results show that strong driving inputs temporarily reset wave dynamics while still facilitating waves over a window of time, including after the stimulus has ended (Figs. 2 to 4). This result is critical as it sheds light on the possibility of stimulus feature integration based on past stimulus history and present stimulus conditions (*7*). Moreover, periods with zero-phase lag (stationary oscillation bump; no wave) could enable short-timescale integration localized to the barrel column, while periods during wave propagation could enable integration over longer timescales and across larger areas (*19*), further influencing sensory integration. In addition, on the basis of their propagation speed, our results (Figs. 2 and 3) suggest that these waves travel along unmyelinated horizontal fibers and have their properties shaped in a context-dependent manner (Fig. 3). Specifically, wave initiation probability was higher across the principal and surrounding barrels when evoked by stimuli associated with a reward, and these waves tended to travel faster than null stimuli, indicating more precise phase gradients (Fig. 3, F to J). Such phase differences combined with our computational results (Fig. 6) point to delicate changes in excitation-inhibition balance (*74*) with feedback-induced inhibition playing a key role in shaping wave-related circuit dynamics (Fig. 6 and fig. S11) (*44*). Physiologically, we demonstrate that traveling wave properties are controlled by wMC-induced network sparsity in wS1 L2/3 (desynchronized asynchronous state) (Fig. 5) and precisely timed action potentials in wS1 L5 (synchronized output) (Fig. 7). In relation to active touch discrimination, our results suggest that wMC feedback to wS1 is more prominent during go trials, possibly due to larger touched-evoked responses in wMC in go versus no-go trials (*75*). Given that wMC feedback modulates both excitatory and inhibitory cells in wS1 and that our computational model highlights the critical role of inhibitory control on network activity (*76*) suggests that traveling wave structure in wS1 reflects translaminar excitatory-inhibitory balance. Specifically, the coupling between sparse waves in L2/3 and precisely timed dendritic regenerative spikes in L5 suggests that a unique “translaminar spacetime code” could help integrate stimuli in wS1. In particular, trials with weak late wave dynamics reflective of weak wMC-induced inhibition and increased L2/3 activity in wS1 could promote network activity dominated by fluctuations and continuous sampling. On the other hand, trials with strong late wave dynamics observed in our study could reflect periods of optimal wMC-induced inhibition in wS1, which leads to higher reliability and a reduction in noise correlations (Figs. 3, 5, and 7) (*77*).

### Translaminar spacetime patterns could help refine cortical coding

Barrel cortex neurons are known to exhibit selectivity to the global direction of the tactile stimulus (*78*). This would imply that individual neurons integrate across space to extract higher-order stimulus features. It would thus seem apparent that traveling waves elicited via touch from one whisker could prime surrounding cortical columns to upcoming stimuli by signaling their reference frame to adjacent somatotopic spaces in the cortex, enabling more collective integration of stimulus features rather than local independent variables (*46*, *79*). Critical to this facet are neurons in the supragranular layers of the cortex that play a key role in orchestrating wave propagation (Figs. 5 and 6). Here, excitatory L2/3 neurons are known to exhibit mixed selectivity with a sparse representation of touch and object location (*80*). These sparse L2/3 patterns likely require feedforward and local recurrent intracortical connections (*11*). Through these intracortical circuits, L2/3 ensembles may integrate tactile features that span multiple whiskers (*46*). We hypothesize that traveling waves enhance the fidelity of sensory-evoked L2/3 ensembles via sparse network activation (*81*). These ensembles, possibly gated (*74*) dynamically by traveling waves, could facilitate the representation of certain stimulus features, improve dynamic range, and aid the classification of multiwhisker stimuli*.* The transformation of the feedforward stimulus in L2/3 should enhance the robustness of feature classification to noise and variations of the input signal to facilitate downstream processing (*82*). In addition to direct feedforward processing, traveling waves controlled via local and long-range recurrence could help link stimulus features across time and contextualize stimuli based on the past information.

The effect of traveling waves on translaminar circuits beyond L2/3 is an open question. Studies in brain slices suggest that L5 neurons play a role in controlling superficial excitation (*83*) and the spread of traveling waves (*84*). The microcircuit that enables this translaminar dynamic likely plays a critical role in shaping cortical output and behavior. Moreover, L5 neurons receive not only direct feedforward drive from L2/3 but also top-down motor feedback onto their apical dendrites. It is thus feasible that traveling waves generated via bottom-up thalamic drive could be organized by translaminar interactions sculpted by long-range feedback. Our recordings support this theory and revealed, in addition to the sensory-evoked early wave, a late wave lasting hundreds of milliseconds poststimulus. The strength of the late wave was time-locked to L5 dendritic Ca^2+^ spikes, nearly abolished by GABA_B_ agonists injected into the calcium-rich zone and strongly modulated by wMC, signifying a role for feedback-driven wave control (Fig. 7). Moreover, L2/3 neurons alone did not exhibit strong modulation during periods of the late wave (Fig. 5 and fig. S12E). This finding asserts that the recorded late wave surface potentials are a reflection of regenerative dendritic spikes occurring a few hundred microns below the pia (*85*)—events which are tightly linked to sensory perception (*27*) and consciousness (*86*). Notably, such large surface potential swings are in line with previous reports (*28*) and is supported by the fact that the dipoles created by the distinct geometry of pyramidal cells have long been considered to dominate field potential recordings captured using electroencephalography and magnetoencephalography (*87*, *88*). Overall, our results suggest that traveling wave dynamics from the cortical surface reflect distinct translaminar circuit features critical for optimal sensory perception.

### Motor feedback organizes traveling waves via changes in Excitation-Inhibition (E-I) balance

Sensory perception is an active process where the brain receives and processes information based on previous experiences, expectations, and current goals. Here, circuits bind and route information across specific pathways over behavioral timescales to create perceptual experience. Within this context, the interaction between sensory and motor cortices is crucial in accurate sensory perception and discrimination and controlling what sensory information the brain will receive (*89*). In the barrel cortex, interactions between sensory and motor cortices are of fundamental importance for accurate sensory perception and discrimination (*47*, *48*, *90*–*92*). wMC projections show a strong preference for deep layers (L5/6) and L1 but are known to also broadly engage different excitatory and inhibitory cell types in sensory cortex (*25*, *66*). Pure excitatory motor cortex feedback to wS1 (*92*) has been reported to enhance spiking responses to touch via supralinear amplification (*93*), encode movement and touch responses (*90*, *91*), drive spiking across cortical layers (*47*), and generate calcium transients in L5 apical dendrites (*26*). On the contrary, motor feedback–induced inhibitory effects have been proposed to vary in strength for the purpose of enhancing sensory coding and reliability (*94*) and have been reported to suppress responses to distractor stimuli in the barrel cortex (*48*).

These sensory-motor interactions occur over timescales of tens to hundreds of milliseconds, suggesting that connections occur over a large loop and are highly recurrent and not purely monosynaptic. It suggests that traveling waves might be able to bind several antecedents, along with past history, and current stimulus conditions. Our data support a model in which spatially restricted motor feedback to wS1 targeting both excitatory and inhibitory neurons enhance sparse coding in L2/3; “sparsifying” the wave (Fig. 6). This is confirmed by our computational results which show mechanistically how this phenomenon can emerge across a wide range of feedback-targeted inhibitory and excitatory ratios (fig. S11), consistent with recent results from local recurrent circuit models signifying a change in the stability point of the network (*11*). Notably, we hypothesize that such feedback-induced changes in wave patterns might be a way to selectively engage ensembles, a form of selectivity within mixed representations (*52*).

Given that wMC inputs synapse onto both L5 and L2/3 neurons (*65*) indicates a critical role of wMC feedback, not only in sparsifying L2/3 but also in sharpening L5. This result is particularly noteworthy as wMC feedback has been shown to elicit calcium transients in L5 apical dendrites, a feature critical for accurate sensory perception (*27*). Our data add to this framework, demonstrating that not only can dendritic potentials be organized into a traveling wave but also show that wMC feedback sharpens L5 dendritic calcium transients. Such an effect can induce synchronized burst firing in L5 somas, leading to heightened downstream transmission for goal-directed sensory discrimination. Our results are also in line with the retinotopic mapping of cortico-cortical feedback circuits recently discovered to engage apical dendrites in visual cortex (*73*), suggesting a generalized role of reverberating waves and dendritic excitability.

Our results are also in line with recent works supporting a translaminar code (*95*, *96*) in which inhibitory microcircuits mediate L2/3-L5 coupling. Specifically, somatostatin (SST) interneurons were shown to be responsible for L5 suppression of touch-evoked activity during optogenetic activation of L2/3 (*95*), highlighting gain control. Other reports have shown that that deep-layer SST interneurons are potent modulators of L5 apical dendritic activity (*96*, *97*). These findings mirror our observations of sparse L2/3 maps sharpening L5 during passive touch in a traveling wave format, which leads to speculate that traveling waves across superficial layers might depend on unique interneuron subtypes.

### Outlook

Our study shows that touch-evoked traveling waves are supported by a unique translaminar spacetime circuit pattern (Fig. 7). We used NeuroGrids, two-photon imaging, silicon probes, pharmacology, optogenetics, and network modeling to establish a connection between the dynamics of traveling waves and the underlying circuit dynamics. Using our custom-fabricated grids, we first showed that whisker touch evoked prominent patterns of traveling waves across the mouse barrel cortex (Figs. 1, 2, and 7). Our data suggest that these touch-evoked broadband waves can be classified into two primary categories: early-onset feedforward waves in the beta-gamma frequency bands and late-onset, feedback-driven waves in the beta-theta bands that are modulated by behavioral context (Figs. 2, 4, and 7). In agreement with previous computational efforts describing how traveling waves shape the cortical landscape, we found that different traveling wave patterns are hallmarked by changes in L2/3 network sparsity (Figs. 5 and 7), establishing the first, to our knowledge, experimental demonstration of the sparse wave theory. We computationally validated these results using a recurrent balanced state spiking neural network model, which revealed that excitatory feedback inputs (for example from wMC) to even a small subpopulation of inhibitory cells within the locally recurrent network (locally in S1) could have widespread effects in sparisying spiking in the cortical circuit (Fig. 6), reflecting a state of inhibitory stabilization (*62*). These results led us to question how a prominent late wave surface potential emerges as sparsity in L2/3 constricts superficial activity. We show that calcium electrogenesis in L5 apical dendrites is strongly implicated in driving the late surface potential (Fig. 7), also establishing a biomarker of dendritic dynamics that one can reliably map from the brain surface. Future studies will investigate the role of L5, and particularly L5A and L5B, in the spread and propagation of traveling waves (*84*). In summary, we found that whisker touch evokes an early-onset feedforward wave, a motor-feedback–dependent late wave, and that this feedback initiates inhibitory action to concomitantly shape L2/3 population sparsity and drives precise L5 dendritic potentials, thus inducing a translaminar spacetime code.

In this work, we did not distinguish between L5 subtypes in contributing to wave dynamics. It is conceivable that wMC feedback sharpens and synchronizes activity between sublayers, heightening both cortico-cortical and subcortical signaling. Another strong possibility is that wMC feedback synchronizes calcium spikes across L5B cells to promote descending activity, which has previously been shown to be a critical component for sensory perception (*27*). Thick-tufted L5B pyramidal neurons show enhanced bursting during active touch in addition to a secondary volley of spiking 50 to 120 ms following touch (*98*), similar to the timescales of the late wave we observed. Critically, one could envision a cortico-thalamocortical loop in which traveling waves gate timing in L5B, which feeds to the thalamus and, in turn, feeds back to L5A. Given anatomical evidence that L5A synapses onto L5B dendrites (*18*) as well as L2/3 (*99*) could set the stage for unraveling precise phase locking across thalamocortical loops and elucidating the role of L5 in controlling superficial wave spread (*84*).

## MATERIALS AND METHODS

Please refer to the Supplementary Materials for information regarding animal subject details, surgical procedures, grid recordings and electrophysiology, viral injections, intrinsic optical imaging, passive whisker stimulation, active touch: lick detection, and two-photon imaging.

### Grid microfabrication

We used conventional photolithography approaches to fabricate 10 to 15 parylene-based surface grids simultaneously on 4″ silicon (Si) wafers. We first cleaned the Si wafers (Nova Electronic Materials) by sonicating in toluene, acetone, and IPA (isopropyl alcohol) for 5 min each. We then dried the wafers with nitrogen and performed a 1-min O_2_ plasma clean (March Jupiter II). For parylene deposition, we used chemical vapor deposition (Specialty Coating Systems) and 3 g of dimer to deposit ~3 μm of parylene C onto four to five wafers simultaneously. To remove leftover monomers from the parylene film, we vacuum-baked the wafers overnight at 140°C.

To form the metal recording sites, leads, and connecting pads, we used photolithography. We spun 300 nm of LOR 3A (lift-off resist) and baked for 10 min at 150°C followed by 3 μm of AZ1518 and a 2-min bake at 100°C. We exposed the photoresist with the first metal pattern with a power of 224 mJ/cm^2^ using a maskless aligner (Heidelberg MLA 150) and developed the wafers for 2.25 min in MF-26A followed by a water rinse. Following development, we performed a brief 10-s O_2_ plasma clean (March Jupiter II). For metal deposition, we used radio frequency (RF) sputtering (PVD SD-400) to deposit 300 nm of Au with a 5-nm Ti adhesion layer. We used deposition rates of ~1.4 nm/min for Ti, 5 nm/min for the first 20 nm of Au (this reduces thin-film stress), and 7 nm/min for the rest of the Au layer. We performed liftoff overnight in Remover PG and rinsed with acetone and IPA. To drive off any leftover solvents, we baked the wafers at 110°C for 5 min.

To reduce the electrochemical impedance of the recording electrodes, we repeated the above photolithography steps with a different mask pattern to deposit nanoporous Au onto only the recording sites (*31*, *32*, *100*). For this sputtering process, we cosputtered Au and Ag simultaneously at dc powers of 50 and 120 W, respectively, to a final metal thickness of 400 nm. Following liftoff, we dealloyed the metal film in 70% nitric acid for 3 min at 70°C. This process etches the Ag in the alloy and leaves behind porous Au. We rinsed the samples in water, dried them with nitrogen, and dehydrated them at 70°C for 3 min and 110°C for 5 min.

To encapsulate the deposited metal, we performed a brief 30-s O_2_ plasma clean to activate the surface of the bottom parylene layer, deposited another 3-μm film of parylene C with the same technique as above, and vacuum-baked overnight at 140°C for a final parylene thickness of 6 μm. We found this ultrathin film to be difficult to handle postfabrication. Therefore, we performed another photolithography step to strengthen the backend of the probe with a layer of 15-μm SU-8. To do this, we again activated the parylene surface with a 30-s O_2_ plasma clean, spun SU-8 2010 onto the surface to a thickness of 15 μm, and baked the wafers for 3 min at 95°C. We used a mask aligner (MA6) to expose the resist with a dose of 160 mJ/cm^2^, baked the sample for 3 min at 95°C, developed in SU-8 developer for 3 min, and rinsed with IPA. To reduce SU-8 stress and improve the SU-8/parylene adhesion, we hard baked the wafers with a ramping bake (2 min at 100°, 110°, and 120°C and 1 to 2 hours at 130°C). This SU-8 layer only supports the backend of the probe (i.e., the large connecting pads and approximately 75% of the probe length), while the grid array portion that sits on the brain remains only parylene and ultraflexible. At no point have we observed delamination between the parylene-parylene or parylene–SU-8 layers, which we attribute to the O_2_ surface activation steps, vacuum baking steps, and temperature ramping.

The final fabrication step on the Si wafer is to etch away parylene to shape the probe and expose the porous Au recording sites and large Au connecting pads (*101*). A 3-μm parylene etch was necessary to expose the recording sites and a 6-μm parylene etch needed to define the outer edge of the probe. We found the simplest approach was to perform the etch steps simultaneously. We spun AZ9620 to a thickness of 15 μm, baked for 4 min at 100°C, exposed with maskless lithography (2100 mJ/cm^2^ exposure), and developed for 15 min in MF-26A. To avoid overetching the recording sites, the mask holes above the recording electrodes were 10 μm in diameter, which expanded to a final diameter of ~25 μm during the etch process. We used a high-power (250 W) O_2_ plasma supplemented with Ar and SF_6_ to etch the parylene layers (March Jupiter II). We avoided overheating of the sample by etching for 1 min followed by 1-min cool-down period of no plasma. We repeated this cycle six to eight times for a total etch time of 6 to 8 min until the Si substrate was visibly clear of parylene. Last, we stripped the etch mask in acetone and rinsed the final wafer product in acetone.

### Grid packaging

To remove the parylene probes from the Si substrate, we submerged the entire wafer in deionized water and used fine-tip surgical tweezers to gently separate the parylene from the underlying wafer. To facilitate this process, the backend of the probe has a “handle” with only parylene and SU-8 layers. This handle allowed us to begin the separation of the parylene and silicon without damaging important metal structures. In our experience, once the separation begins, the parylene probes cleanly separate from the Si substrate. We also note that the SU-8 thickening layer facilitates parylene removal and found it difficult to remove probes constructed of only 6-μm parylene. Furthermore, we anecdotally note that probe removal soon after fabrication (<24 hours) markedly reduced probe curling.

After removal and drying, we bonded the backend of the probes to a laser-cut 0.010″-thick polyether-ether-ketone film (*30*) (CS Hyde, 37-10F-24″ × 24″) using a 20% mixture of polyvinyl alcohol in water (Sigma-Aldrich, 341584-25G). This film allowed for stable insertion of the probe backend into a 32 Ch ZIF clip [Hirose, FH12-32S-0.5SH (*55*)], surface mounted to a custom 1.0 × 0.5″ printed circuit board. Onto the polychlorinated biphenyl, we also mounted a ground pin and 32 Ch Omnetics connector (NPD-36-VV-GS), which allowed us to interface the parylene grids with a 32 Ch Intan amplifier (RHD 2132).

### Active touch: Training and recordings

For the active touch paradigm, we replicated previous work demonstrating a pneumatic system that extends pistons into the whisking range of head-fixed animals (*46*). The organization of a single trial can be found in fig. S5A. Briefly, animals must reach a running threshold of 5.6 cm/s before the go or no-go stimulus extends into the whisking range for an active sampling period of 1.3 s. For the go stimulus, the animal must lick within this window. If successful, then a water reward is delivered and an intertrial interval of at least 4 s is used. For the no-go stimulus, animals must withhold a lick. If the animal correctly rejects the no-go, then an intertrial interval of 4 s is used. If the animal licks, then the trial is considered a false alarm and an intertrial timeout of 14 s initiates. To control the behavioral state of the animal, before the next trial initiates, the animal must again reach the running threshold. Throughout training and recordings, we used white noise to drown out sounds from the pneumatic parts and water delivery system.

For discrimination training, we began with the headplate surgery. Animals were given 2 to 3 days for headplate habituation and recovery. Mice were then head-fixed and allowed to habituate to the running wheel for 3 to 4 days for 1 hour every day. These mice were then water restricted, and their whiskers were trimmed to leave C1 and D1 whiskers on the right whisker pad. We then introduced the go stimulus (C1 piston) and trained animals on classical conditioning for 3 to 4 days to form the association between C1 touch and water reward. After this period, we introduced the no-go stimulus (D1 piston) until mice showed anticipatory licking for the go stimulus (3 to 4 days). Last, mice were moved to operant training where they learned to withhold their licking for the no-go stimulus (>5 days). Training every day for 1 hour was maintained until these mice reached a discrimination index, *D*′, greater than 1.5 for at least two consecutive sessions (fig. S5B). We calculated *D*′ with the following equation

D′=Z(hit rate)−Z(false alarm rate)

where *Z* is the inverse cumulative distribution function of gaussian distribution.

One to 3 days before recordings, the C1 and D1 whisker barrels were identified through intrinsic imaging. On the day of the electrophysiology recording, mice were briefly (20 to 30 min) anesthetized to perform a craniotomy over the identified barrels. The exposed area was covered with silicone gel (Dowsil) and Kwik Cast silicone (World Precision Instruments). We allowed animals a minimum of 3 hours of recovery before beginning recordings. For electrophysiology during the discrimination task, mice were placed in the experimental rig, and grids were lowered over the go and no-go barrels to record neural activity while the mouse discriminated between the presented stimuli. Note that while the grid was placed as evenly as possible over each barrel and contact was successfully made with each barrel for all recordings (as determined by the vasculature and intrinsic imaging), the precise grid position from animal to animal changes due to natural changes in anatomy the grid placement angle. We performed recordings in five animals. However, one animal was removed from the discrimination analysis due to an experimental issue that led to falsely detected licks. The data for this animal were included when analyzing the effects of whisking and touch on traveling waves (fig. S5, C and D), but was removed for analyses comparing hit and correct rejection trials (Fig. 3 and fig. S5F).

### Active touch: Whisker tracking and kinematics

A high-speed infrared camera (Photon focus DR1) was used to track whisker kinematics at 500 fps during the recording session. Camera video frames were recorded on Intan 512 controller and synchronized with neural data via external triggers generated by a National Instruments card. For postprocessing, DLC was used to track whisker movement and obtain touch times (*49*). For the DLC training dataset, we manually labeled 200 video frames for each animal. Four labels were evenly spaced to mark each whisker in a video frame. The DLC neural network was trained for 360,000 iterations, and the final labels were manually checked for accuracy. The whisker position was calculated for each label for both the whiskers with reference to a user defined point on the whisker pad. Whisker phase was bandpass-filtered between 1 and 30 Hz. The whisker bend was calculated from the three distal labels on each whisker using Menger curvature (*c*).

$$c=\frac{4A}{|x-y||y-z||z-x|}$$

where *x*, *y*, and *z* are the coordinates of three labels on a whisker, and *A* is the area of the triangle formed by these three labels. Whisker touch frames were identified from when the whisker label enters a region of interest (ROI; manually marked around the touch surface).

### Optogenetics

All optogenetic light stimulation was performed with a fiber-coupled light-emitting diode (LED; 200-μm-diameter core) placed on either the surface of the brain or surface of thinned skull. ChR2 was activated with a 470-nm LED (Thorlabs, M470F3, 7 mW) and NpHR with a 595-nm LED (Thorlabs, M59F2, 4 mW).

For wS1 L5 ChR2 activation (Fig. 7B), the LED was placed at an approximately 35° angle to the silicon probe. We reduced the light artifact on the silicon probe by facing the recording electrodes away from the light direction and offsetting LED stimulation by ~200 μm laterally from the probe insertion sight so that the maximum light intensity did not hit the backside silicon. For these experiments, we also used the analog output of an Arduino to ramp the LED power to reduce the photo artifact (*102*). We ramped the LED power up for 15 ms to the maximum power, held the maximum power for 5 ms, and then ramped down for an additional 15 ms for a total stimulation time of 35 ms. This method reduced but did not abolish the light artifact. We used a trial-to-trial interval of 10 s.

For wMC ChR2 activation during wS1 silicon probe recordings (fig. S13I), the LED was placed perpendicular to the brain surface, and brief square-wave pulses of 1, 5, or 10 ms were delivered. We used a trial-to-trial interval of 10 s. For wMC NpHR inhibition (fig. S13, F to H), the LED was placed at an approximately 35° angle to the surface of wMC, which allowed us to vertically insert a silicon probe in a subset of animals to validate single-unit inhibition. For these experiments, we turned on the LED 1 s before whisker touch, and the light remained on for another second after touch. We interleaved trials with the LED on and off to observe the effects of wMC inhibition on the late wave. We used a trial-to-trial interval of 10 s.

### In vivo pharmacology

For in vivo pharmacology, we pulled glass micropipettes to a diameter of ~20 μm and attached the puff pipettes to a pneumatic microinjector (Applied Scientific Instrumentation, MPPI-3). All injections were performed with a series of 10-ms puff pulses spaced 1 to 2 s apart. For wS1 baclofen (Fig. 7C), we placed grids over the C1 barrel and navigated the puff pipette to the small opening in the grid center. We used a 50 μM baclofen solution dissolved in fresh artificial cerebrospinal fluid (ACSF) and injected 20 to 50 nl at 10 nl/min at a depth of 250 μm. Immediately following injections, we recorded data for 30 min with a trial-to-trial interval of 15 s to observe the effects of baclofen. Data recorded 30 to 90 min postinjection was considered the “recovery” period, when we observe the effects of baclofen to dissipate. We conducted baseline recordings before baclofen injection. As a control, we injected ACSF at the same depth and rate either before the baclofen injection or after the recovery period. For wMC muscimol (Fig. 7D), we used a solution (5 μg/μl; Sigma-Aldrich, M1523-10MG) dissolved in fresh ACSF and injected 250 μl at 20 nl/min at a depth of 500 μm. We recorded the effects of muscimol up to 4 hours postinjection. As a control, we injected ACSF-only at the same rate and depth before muscimol.

### Traveling wave simulations

We adapted a large-scale spiking network model introduced in previous work (*4*). The model is composed of leaky integrate-and-fire neurons, with 80% excitatory and 20% inhibitory, operating in a balanced state (*103*) characteristic of the barrel cortex in vivo (*104*, *105*). Neurons in the model are arranged on a grid, with up to 450,000 neurons distributed over a 4 mm–by–4 mm area, with periodic boundary conditions. A large number of synapses per cell (1000) allow synapses in the model to approximate the scale observed in vivo (*106*), in contrast to models with smaller numbers of synapses per cell, where recurrent synapses need to be stronger and correlations are overexpressed (*107*). Axonal conduction delays increase linearly with distance between pre- and postsynaptic cells

$$\tau_{\mathrm{ij}}=\tau_{s}+\frac{d_{\mathrm{ij}}}{\vartheta_{c}}$$

where τ_*ij*_ *I* is the delay from neuron *i* to neuron *j*, and τ_*s*_ is a fixed delay accounting for synaptic vesicle release, *d*_*ij*_ is the Euclidean distance between neurons *i* and *j*, and ϑ_*c*_ is the axonal conduction speed. This network generates self-sustained activity with spontaneous spiking fluctuations consistent with the asynchronous-irregular regime (*103*, *108*). A simulated LFP is calculated from summed excitatory and inhibitory synaptic activity in adjacent, nonoverlapping pools of 10 × 10 neurons (corresponding to 67.8 μm × 67.8 μm) (*109*) and is compared to the LFP in the electrophysiological recordings. Simulations were performed using custom software programmed in C (NETSIM; http://mullerlab.github.io). Equations in the model were integrated using the forward Euler method with a time step of 0.1 ms. We then studied the effect of excitatory feedback projections on traveling waves generated by this network. Feedback projections were modeled as external Poisson inputs delivered for a short time window (20 ms), while an individual wave propagates across the network. We focused on feedback delivered only to a local patch of the network and compared the dynamics in the local network during stimulation to the dynamics before stimulation and in the network outside this local patch. Next, we studied how varying the probability with which the feedback projections target excitatory or inhibitory neurons increases the sparsity of neuronal spiking while the wave propagates across the network.

### Analysis: Data inclusion criteria

For each animal, we recorded 50 to 300 passive touch trials. During postprocessing, we considered a trial to be “responsive” if a detectable LFP was observable within 70 ms of touch. We defined a detectable LFP as a touch-evoked signal with a maximum at least three times greater than the prestimulus SD (500-ms window before touch). All further analyses were performed on these “touch-responsive trials,” which typically consisted of >80% of trials.

### Analysis: GP

We used the GP approach to find the instantaneous phase of both the wideband- and narrowband-filtered data (*5*). We filtered all data with a fourth-order Butterworth filter (MATLAB’s *filtfilt* function) in the relevant frequency bands and down-sampled the LFP to either 2500 or 1000 Hz. To attain the phase of the LFP signal, we used the GP method for calculating the analytic signal. This technique is described in depth elsewhere (*5*) and summarized here. This technique is very similar to existing traveling-wave analyses that leverage narrowband filtering (*1*, *3*, *13*, *20*). This approach yields a real-valued, filtered LFP signal, θ(*x*, *y*, *t*), and uses a Hilbert transform to calculate the analytic signal Χ

$$X\left(x,y,t\right)=V\left(x,y,t\right)+\mathrm{iH}\left[V\left(x,y,t\right)\right]=A\left(x,y,t\right)e^{i\varphi \left(x,y,t\right)}$$

where Χ is the analytic signal, *V* is the LFP, *H* is the Hilbert transform, *A* is the instantaneous amplitude, φ is the instantaneous phase, and *x* and *y* are the electrode location and the computation occur at each time point *t*. Typically, the Hilbert transform is calculated using a single-sided fast Fourier transform (FFT; see MATLAB’s *hilbert* function). In the analytic signal, sinusoids in the LFP are represented in the complex plane as circular contours, and the instantaneous phase can then be calculated from the arctangent function (MATLAB’s *angle* function). From the instantaneous phase, the instantaneous frequency can be calculated as the time derivative

$$f\left(x,y,t\right)=\frac{\partial \varphi }{\partial t}$$

Fast fluctuations in the real LFP signal, *V*(*x*, *y*, *t*), can drive marked changes in the instantaneous phase, φ(*x*, *y*, *t*), and lead to discontinuities in *f*(*x*, *y*, *t*). Narrowband filtering circumvents these problems, and if discontinuities do occur, then the most common method of removing these frequencies is to set them to zero (see MATLAB’s *hilbert* function).

The GP approach can be applied to wideband-filtered LFP by solving this discontinuity problem. Like a conventional wave analysis method, the GP approach first calculates the single-sided FFT of the filtered LFP. The GP algorithm then accounts for frequency discontinuities through a shape-preserving interpolation of φ(*x*, *y*, *t*), yielding an instantaneous phase that matches the large signal fluctuations at a given point in time, rather than smaller, fast frequencies. The toolbox for performing the computation is publicly available (https://github.com/mullerlab/generalized-phase).

### Analysis: Traveling wave detection with GP

After calculating the instantaneous phase of either the wideband or narrowband LFP with the GP method, we detected traveling waves by analyzing the circular-linear correlation between phase and distance. These methods are outlined in fig. S2. First, we detect crossings of −π/2 in the phase domain (i.e., positive-rising potentials in the LFP) and label these as “evaluation points” where traveling waves potentially exist (fig. S2A). For these evaluation points to be categorized as a true traveling wave, the phase delays across the grid must follow a linear pattern outward from a source point (i.e., the initiation point of the wave). We found this putative source using functions in the Wave toolbox (*110*) (https://github.com/mullerlab/wave-matlab), which finds the point of maximum divergence of the phase across the grid (fig. S2B)

D(x,y,t)=∇·∇φ(x,y,t)

where φ(*x*, *y*, *t*) is the phase across the grid at time point *t*, ∇ φ(*x*, *y*, *t*) is the phase gradient, and ∇ · ∇ φ(*x*, *y*, *t*) is the divergence of the phase gradient, *D*(*x*, *y*, *t*). The putative source point, *S*, of the wave is the point of maximum divergence

$$S={\text{argmax}}_{x,y}\left[D\left(x,y,t\right)\right]$$

The electrode location at the source point *S* is then considered the site of wave initiation. Moving outward from this source point, a traveling wave should exhibit a linear increase in phase with distance. A constant phase or phase that is uncorrelated with distance is not considered a traveling wave. We calculated this correlation using the circular-linear phase versus distance correlation using the CircStats toolbox (*111*), which yields a correlation value between 0 and 1 for all potential waves. Waves with a high correlation are considered true traveling waves (fig. S2C). To determine the correlation threshold for traveling waves, we created a “null distribution” of correlation values by randomly shuffling the electrode locations across the grid. We ran our GP pipeline on this shuffled data to detect evaluation points (i.e., pseudo-traveling waves) and measure.d the phase versus distance correlation values for each point. Iterating through this shuffling process formed a large null distribution low correlation value. We used the 99th percentile of the distribution as a threshold for traveling wave detection in unshuffled LFP (fig. S2C). This process was performed for each animal and a unique threshold used for each animal, usually between 0.5 and 0.6.

The phase versus distance (i.e.,φ versus *x*) relationship for each traveling wave yields valuable information. The wave number *k* follows from the spatial derivation

$$k=\frac{\partial \varphi }{\partial x}$$

in units of radians per meter. The instantaneous frequency is the time derivative

$$f=\frac{\partial \varphi }{\partial t}$$

in units of hertz. The instantaneous angular frequency is

ω=2πf

in units of radians per second. Last, the wave speed can be calculated

$$v=\frac{\omega }{k}$$

in units of meters per second.

The GP pipeline labels the wave onsets (i.e., −π/2 phase crossings), which are the positive LFP upswings of the wave. To find the amplitude of each wave, we found the LFP amplitude of the next zero-phase crossing point, which labels the LFP peaks.

### Analysis: Late wave detection and amplitude

To sort trials based on the strength of the late wave, we first normalized the single-trial LFP to the mean of the 500-ms prestimulus period. We considered the baseline of the recording to be the SD of the prestimulus period. We then found the amplitude of the late wave in a window 80 to 200 ms posttouch. If this amplitude exceeded 3 baseline SDs, then we considered the trial to have a detectable late wave. Trials under this threshold were categorized as weak late wave trials. Because of the small variation of the late wave across the grid, we only performed this sorting using one representative electrode chosen by the largest LFP amplitude. We found similar sorting results when using the LFP power rather than the amplitude.

To measure the average amplitude of the late wave, for each channel, we averaged the LFP across all touch-responsive trials in the data. Using a custom MATLAB GUI, we plotted these waveforms, annotated the approximate onset of the late spike, and then ran an algorithm that automatically detected the late wave onset (the trough) and the late wave peak (the maximum 100 to 300 ms after touch). The late wave amplitude for the channel is then computed as the trough-to-peak amplitude (see fig. S13B).

### Analysis: Removing two-photon scanning artifacts on NeuroGrids

Resonant laser scanning during two-photon imaging led to mild noise artifacts on the NeuroGrid data [see fig. S1 (D and E)]. To remove these artifacts, we filtered the LFP data with multiple sixth-order Butterworth notch filters at frequencies of 30 Hz and its harmonics up to 360 Hz. This approach removed the majority of the noise power while preserving the overall LFP (fig. S1E).

### Analysis: Two-photon calcium imaging segmentation

Each two-photon imaging trial generated a 32-bit TIFF stack. For each animal, we concatenated all stacks using ImageJ, denoised with a Kalman stack filter plugin (using default parameters), and converted the concatenated files to eight-bit format. We used a CaImAn pipeline to segment ROIs and extract calcium traces from two-photon imaging data (*112*). Because of our large datasets, we used the “patches” functionality, which analyzes smaller patches of the full FOV in parallel. This pipeline includes motion correction using NormCorr, segmentation using constrained nonnegative matrix factorization, neuropil subtraction, and deconvolution to extract calcium events. Last, features were refined through a convolved neural network classifier. We determined two parameters that were key to dendritic segmentation. We lowered the classifier threshold (“cnn_thr”) from 0.4 to 0.3 and the NMF converging coefficient from 0.8 to 0.5. We found that the patches functionality not only labeled neurons well but also provided a number of extraneous ROIs. We manually curated the segmentation post hoc and removed these erroneous ROIs with a custom MATLAB GUI.

We classified cells as touch-responsive if the averaged touch-evoked Δ*F*/*F* transient showed a maximum value three times larger than the baseline SD within 2 s of touch. To calculate the touch-evoked onsets of each cell on a single-trial basis, we first calculated an event threshold for each cell using the 99th percentile of all events in the deconvoluted data. Events that exceeded this threshold were considered true calcium events. We then determined whether an event occurred within a 1-s window after each touch stimulus. If an event occurred, then the largest calcium event within the window in the deconvoluted data is maximum slope of the Δ*F*/*F* upswing (Fig. 7G). We found the onset of the event by finding the preceding inflection point in the deconvoluted traces (Fig. 7F).

### Analysis: Calcium data vectorization, reactivation index, and ensemble detection

Our ensemble analysis pipeline is outlined in fig. S9 and follows previous reports (*54*, *113*). Changes in fluorescence and convolved spiking activity were filtered by omitting events below 2.5 SDs of the noise floor. This ensured robust spiking analysis for neuronal population networks. We first generated a *N* × *T* calcium activity matrix by plotting the mean centered fluorescence values for all detected cells, where *N* denotes the number of neurons and T denotes the total number of frames for each video

$$F_{\text{centered}}=\frac{F-F_{\text{avg}}}{F_{\text{max}}-F_{\text{min}}}$$

We then used a coactivity threshold to transform this calcium activity matrix into *N* × *T* binary spike matrix (fig. S9A). Each row (*N*) represents the spiking events of a neuron. The threshold was determined by shuffling the data (see below) and essentially removing solitary calcium events, focusing our analyses on periods of coactivity. We then computed a vectorized form of the binary matrix by binning frames every 300 ms and summing the calcium events across time in each bin (fig. S9A). This summation produced a matrix of time vectors, *t*_*i*_ = (*N*_*i*_, *T*_*i*_), where *N*_*i*_ is the summed event spikes of time bin *T*_*i*_. This vector series represents temporal changes of grouped neurons.

This vectorized form of calcium activity is advantageous, since now vector-based methods can be used to for correlating changes of network states between vector pairs *t*_*i*_ and *t*_*j*_ using a cosine similarity index (fig. S9B)

$$\text{Similarity index}\;\left(\text{SI}\right)=\frac{t_{i}\cdot t_{j}}{\left\|t_{i}\times t_{j}\right\|}$$

This yielded a nonnegative value corresponding to the cosine angle between any vector pair in vector space (fig. S9B). A value near 1 corresponds to vectors with nearly identical activity patterns within the binned time frame. Running this calculation for all vector pairs yields a *T*_*i*_*xT*_*i*_ “similarity map” that indicates periods of similar patterns of activity across the FOV (fig. S9B). We then further refine this similarity map by removing all vectors that do not show a statistically significant similarity with any vector pairs (threshold determined by shuffling the data), which we refer to as the reactivation index as this metric indicates the prevalence of similar reactivation patterns across the imaging trial.

The similarity map for an animal highlights periods of similar activity patterns during the recording. To identify notable patterns in the matrix, we found the principal components of the high-dimensional data using singular-value decomposition (SVD). SVD allows for the identification of components that contribute the most to a given network state. The SVD of a matrix can be represented as

$$M=U\sum V^{T}$$

where *M* is the similarity matrix, *U* and *V* are the orthonormal bases, and ∑ are the eigenvalues of the principal components in matrix *M*. In our case, because *M* is symmetric (see fig. S9B), this reduces to

$$M=V\sum V^{T}$$

We found the eigenvalues that captured the majority of the data variance by comparing the SVD to a shuffled dataset. This typically left 3 to 10% of eigenvalues per animal that capture >90% of the data variance. Activity vectors are then grouped and spatially projected using these eigenvalues and reproduce the main activity patterns of the original data to a high degree. These eigenstates are used as candidates for neuronal ensembles.

Each eigenstate (i.e., activity state) is a vector composed of the activity of all neurons within the FOV. However, only a subset of neurons within this vector is coactive and form the majority of the activity within the eigenstate. These active cells compose the neuronal ensemble. To find these active cells, we used a SØrenson-Dice correlation (SDC) to quantify synchronous firing events of individual neurons (fig. S9C). Unique neuronal pairs identified through the binary matrix (*N* × *T*) are linearized and filtered through a function

$$\text{SDC}=\frac{1}{n_{\text{max}}}\sum \left[S_{i}>0.25\right]\in \left[P\right]=\left\{\begin{array}{c} 1\;\text{if}\;P\;\text{is true},\\ 0\;\text{if}\;P\;\text{is false} \end{array}\right\}$$

The resulting correlation matrix represents event pairing of identified neurons throughout the video and yields critical factors such as node strength (i.e., a measure of activity), functional connectivity strength between neuron pairs, and numbers of functional connections (fig. S9C). These attributes both identify the neuronal ensemble that composes the activity state and also yields network functional connectivity metrics. The proportion of neurons within an ensemble that were coactive above shuffled chance was defined as the ensemble reactivation.

To set ensemble detection thresholds, multiple data shuffling procedures were performed. Initial spikes per neuron in the binary matrix *N* × *T* were temporally shuffled across each frame of the time series. An event shuffle is further introduced between two temporally shuffled ROIs to produce a spatiotemporal shuffled binary matrix. This binary surrogate is repeatedly shuffled. Shuffling was done by generating a random permutation equal to the length of the neurons and frames. Spikes were then indexed to each random number generated. This method randomizes the network state rendering the population of the spiking as stochastic events.

### Analysis: Single-unit recordings

We preprocessed data by bandpass filtering with a fourth-order Butterworth filter between 300 and 3000 Hz (MATLAB’s *filtfilt*). After filtering, we subtracted the median signal across all channels from each recording site (i.e., common median referencing) to remove shared high-frequency noise (*114*). We used KiloSort2 for spike sorting silicon probe recordings (https://github.com/MouseLand/Kilosort/releases/tag/v2.0) and manually curated the clustered data using Phy2 (https://github.com/cortex-lab/phy/) (*115*)

### Analysis: Current source density

We used CSD analysis to determine current sources and sink across a linear silicon probe during LFP recordings (*116*). The CSD is calculated from the second spatial derivative the LFP transients

$$I_{m}\left(x,y,z\right)=\left(\sigma_{x}\frac{\partial^{2}V}{\partial x^{2}}+\sigma_{x}\frac{\partial^{2}V}{\partial y^{2}}+\sigma_{x}\frac{\partial^{2}V}{\partial z^{2}}\right)$$

where *I*_*m*_ is the CSD, *V* is the LFP, and σ are the spatial conductivities of the extracellular space. When assuming laminar uniformity and constant conductivity, the derivatives in the *x* and *y* directions become zero, and this calculation can be approximated as

$$I_{m}=\frac{\partial^{2}V}{\partial z^{2}}\approx \frac{V_{n+1}+V_{n-1}-2V_{n}}{{\left(2∆z\right)}^{2}}$$

where *I*_*m*_ is the CSD, *V*_*n*_ is the LFP on the *n*th electrode, and ∆ *z* is the electrode spacing (*117*). For our calculations, we used *n* = 2 and added two Vaknin electrodes (*118*) at the top and bottom of the silicon probe to estimate the CSD across the full shank length.

### Analysis: Spectral analysis of grid and shank recordings

To calculate LFP spectrograms from NeuroGrid recordings (Fig. 4C) and shank recordings (Fig. 4E), we chose a representative electrode and used a continuous wavelet transformation (MATLAB’s *cwt* function) to calculate the single-trial spectrogram between 0.1 and 100 Hz. The single-trial spectrograms for all touch-responsive trials were then averaged to generate the average spectrogram.

To calculate the beta/gamma and theta/gamma power ratios from NeuroGrid recordings (Fig. 4D), we first calculated the average spectrogram from a representative channel as outlined above. We then summed all the data points in the spectrogram in the respective theta, beta, and gamma bands to get the “total power” in the early (0- to 60-ms window) and late (100- to 200-ms window) wave periods. The ratios of the total power yielded the relative beta/gamma and theta/gamma power during the early and late wave periods.

To calculate the relative power profile in laminar shank recordings (fig. S8B), we mirrored previously reported methods (*53*). Because the UCLA silicon probe recording electrodes are organized into a hexagonal format and offset in the *x* axis, we limited our analyses to the linearly aligned electrodes on either the left- or right-hand side of the shank. First, we focused on a time window between spanning −100 ms to +200 ms around the touch stimulus. For each laminar electrode, we then calculated the average power spectral density across trials (MATLAB’s *pwelch*). We then concatenated the average PSD vectors for every laminar electrode into a *f* × *N* matrix where *f* is the PSD frequency and *N* is the number of channels organized from superficial electrodes to deep electrodes. The relative power at each frequency point is calculated by normalizing each column of the matrix to its maximum value. This process can be summarized with the following equation

$${\text{Relative power}}_{n,f}=\frac{{\text{Power}}_{n,f}}{\text{max}\left({\text{Power}}_{1:N,f}\right)}$$

where *n* is the electrode channel, *N* is the total number of electrodes, and *f* is the frequency (*53*).

### Statistical analyses

All statistical testing was performed in MATLAB. For boxplots, the upper/lower edges of the box represent the upper/lower quartiles of the data. The line within the box represents the median of the data. Data points that fall outside the boxplot upper/lower “whiskers” are considered outliers. We tested data for normality using a Kolmogorov-Smirnov test. In all datasets within this study, we determined at least one group per comparison to be nonnormally distributed, and therefore, we used only nonparametric statistical tests. Statistical details can be found within the legends of each figure. In short, we used a rank sum Wilcoxon test (otherwise known as a Mann-Whitney *U* test) to compare unpaired, nonparametric data between two groups. We used a signed-rank Wilcoxon test to compare paired, nonparametric data between two groups. We used a Kruskal-Wallis test to compare unpaired, nonparametric data between three or more groups and followed this with a post hoc Dunn-Sidak test to correct for multiple comparisons. Last, we used a Friedman’s test to compare paired, nonparametric data between three or more groups and followed this with a post hoc Dunn-Sidak test to correct for multiple comparisons. In some cases, we used bootstrapping to create data distributions to compare two conditions. In fig. S2E, we performed 10,000 iterations, randomly choosing 50-ms intervals in the prerun and postrun intervals and quantified the number of detected waves in each interval. In Fig. 7G, we randomly chose 100 dendrites (with resampling) and calculated the SD of Δ*F*/*F* onset times. We repeated this process 10,000 times for the late wave and weak late wave conditions to form a distribution SD values for each condition to compare with a rank sum Wilcoxon test. In fig. S12C, we randomly selected 20 trials with and without the late wave, calculated the mean LFP, calculated the CSD, and then measured the magnitude of the late L2/3 current sink. We repeated this process 50 times for each animal to form distributions of the current sign magnitudes with and without the late wave. Reported *n* values and *P* values can be found within the test and legends for each data comparison.
