# Supplementary material for: Touch-evoked traveling waves establish a translaminar spacetime code
Source: Sci Adv. 2025 Jan 31;11(5):eadr4038. doi: 10.1126/sciadv.adr4038 (PMC11784861; doi:10.1126/sciadv.adr4038)
Supplement: Supplementary file 1 — Supplementary Materials and Methods Table S1 Figs. S1 to S13 [file sciadv.adr4038_sm.pdf]

Science Advances
AAAS

Supplementary Materials for

Touch-evoked traveling waves establish a translaminar spacetime code

Daniel L. Gonzales *et al.*

Corresponding author: Krishna Jayant, kjayant@purdue.edu

Sci. Adv. **11**, eadr4038 (2025)
DOI: 10.1126/sciadv.adr4038

This PDF file includes:

Supplementary Materials and Methods
Table S1
Figs. S1 to S13

Materials and Methods (extended)

Animal subject details

All experimental procedures were conducted in accordance with the guidelines set forth by the NIH, Purdue Institutional Animal Care and Use Committee (IACUC), and the Purdue Laboratory Animal Program (LAP) (approved protocol number 1910001968). All experiments were conducted in adult mice ages 3-8 months old. For passive touch recordings, we used mice with a C57BL/6J (The Jackson Laboratory, #000664) background kept on a 12-hour light/dark cycle in conventional housing with unrestricted access to food and water. Male and female mice were used in approximately equal numbers. For L2/3 imaging experiments, we used Thy1-GCaMP6s transgenic mice (Jackson, C57BL/6J-Tg (Thy1-GCaMP6s) GP4.3Dkim/J, #024275). For experiments with targeted L5 expression, we used Rbp4-Cre mice (MMRC, B6.FVB(Cg)-Tg(Rbp4-cre)KL100Gsat/Mmucd, #037128-UCD). For active-touch experiments, we used EMX1 mice (Jackson, B6.129S2-Emx1tm1(cre)Krj/J mice, #005628) outcrossed with CD-1 mice (Jackson, #022) for multiple generations. We used both sexes maintained in reverse light-dark cycle (12:12 hr.). Behavioral training and recordings were conducted during the animal's subjective night.

Surgical procedures

Mice were first deeply anesthetized with 3 to 4% isoflurane. During surgery, anesthesia was maintained at 1 to 1.5% isoflurane with an oxygen flow rate ~0.1 L/minute. An infrared warming pad (Kent Scientific) was used to maintain the body temperature. Carprofen and Dexamethasone (0.6mg/kg body weight) was injected subcutaneously, and lidocaine injected under the scalp after the induction of anesthesia. Eye ointment was applied, and the scalp shaved and sanitized before the incision. After scalp removal, we briefly applied 3% hydroperoxide to remove excess tissue and immediately cleaned the skull with saline perfusion. Vetbond (3M) was used to seal the tissue surrounding the skull region. We then used dental cement to adhere a custom-designed titanium headplate (Parkell) to the skull centered over the left barrel cortex (1.5 mm posterior to bregma and 3.5 mm lateral to midline). Once the cement solidified, we used a dental drill to partially thin an ~5 mm area of bone around the barrel cortex until the vasculature was clearly visible when soaked in saline. We then dried the skull, sealed the exposed bone with Vetbond, and covered the entire area with Kwik Cast silicone (World Precision Instruments).

Animals were given 3-7 days to habituate to the headplate. 1 day prior to recordings, we trimmed all but the C1 whisker and performed intrinsic optical imaging to locate the C1 barrel (described below). On the day of recordings, the anesthesia and subcutaneous injections described above were performed again. For a ground electrode, we performed a small craniotomy in the forebrain and cemented in place an Ag/AgCl wire inserted between the dura and skull. To access the barrel cortex, we used a dental drill to perform a 2-3 mm cranial window (surrounded by and additional ~2 mm region of thinned skull) centered on the C1 barrel. We regularly perfused the surgical area with clean ACSF. For all grid recordings, we also performed a durotomy with a dura hook (Fine Science Instruments, 10032-13). For experiments requiring only a silicon probe, the cranial window was <1 mm in diameter with a 2-3 mm area of thinned skull around the window. Upon completion of the surgery, we covered the cranial window with a small piece of surgical foam (Surgifoam) soaked in ACSF and transferred the animal to the recording rig where it remained head-fixed for the duration of the experiment.

Grid recordings and electrophysiology

To place the grids over the C1 barrel, we mounted the fully-packaged probe (grids, custom PCB, head-stage) onto a manual micromanipulator. After the craniotomy and durotomy, we transferred the animal to the recording rig for head-fixation. While still lightly anesthetized, we removed the surgical foam and filled the area above the cranial window with ACSF. We then placed the grids on the ACSF, which were held in place through surface tension. Slowly, we aspirated the ACSF, while adjusting the micromanipulator to maintain the grid position over the barrel. Upon initial contact we with brain, we found that the grids did not strongly adhere and could easily be removed through the addition of more ACSF. To promote adhesion, we maintained a very thin film of ACSF over the grids and brain, aspirated the majority of the ACSF (without completely drying the brain), re-wetted the surface with another thin film of fresh ASCF. We repeated this process every ~5 min over the course of ~30 min. We found that this simple protocol led to strong adhesion between the grids and brain such that a pool of ACSF no longer lifted the grids off the brain. For experiments where only surface electrophysiology was required, we placed surgical foam soaked in ACSF over the grids. For experiments requiring grids in combination with pipettes or silicon probes, we maintained a layer of ACSF over the cranial window for the duration of the experiment and gently replenished this solution every ~30 min. Finally, for experiments requiring grids with two-photon imaging, after placing the grids onto the brain surface, we aspirated ACSF to a thin layer, pressed a 3 mm coverslip (Warner Instruments, 64-0720) onto the grids and brain with a micromanipulator, and cemented in place. In most cases, we carefully cemented only the areas of the coverslip not in contact with the grids (see **Figure 1** schematic), which allowed us to recover the parylene probe after recordings.

All electrophysiological data was acquired with Intan Technologies head-stages (32 and 64 Ch) interfaced to an RHD Recording System using RHX Data Acquisition Software. The sampling rate was 20 KHz. For grid recordings with simultaneous depth recordings, we used Cambridge Neurotechnologies H5 64 Ch linear probes (800 um electrode span, 25 µm vertical spacing between electrodes) or H8 64 Ch two-shank probes (250 µm shank spacing, 930 µm electrode span, 30 µm vertical spacing between electrodes). These ultra-thin silicon probes were optimal for insertion through the 50 um-wide slit through the center of our surface grids. For experiments requiring only silicon probes recordings, we used UCLA 64D silicon probes electroplated to an electrochemical impedance of ~300 Kohm (1.050 mm electrode span, 25 µm vertical spacing between electrodes).

Viral injections

We performed injections 4-6 weeks in advance. All anesthesia and surgical protocols were identical to the above protocols. However, we made a small incision in the scalp rather than completely removing the skin. We then made a small burr hole in the skull using a dental drill. An injection micropipette (Sutter BF100-50-10, Sutter P-1000 puller) with a diameter of <50 µm and backfilled with mineral oil was mounted to a microinjector (World Precision Instruments, Micro4 controller, UMP3 micro-syringe pump) for injections. For all injections, we inserted the pipette ~100 µm past the target depth, then slowly raised the pipette up to the target depth. All injections were performed at a rate of 20 nL/min and we waited 5-10 min after injection completion before slowly removing the micropipette. For wS1, we used stereotactic coordinates 1.5 mm posterior to bregma and 3.5 mm lateral to midline. For wMC, we used stereotactic coordinates 1 mm anterior to bregma and 1 mm lateral to midline. Following injections, we sutured the scalp and allowed the animal to recover in a heated cage. The AAV, titer, volume injection, and injection depth are in **Table 1**.

Table 1. Virus injection details

Experiment	Animal line	Plasmid	Addgene#	Titer (vg/ml)	Volume (nL)	Depth (μm)
wS1 L5 apical dendrite imaging	Rbp4-cre	pAAV-hSyn1-Flex-mRuby2-GSG-P2A-GCaMP6s-WPRE-pA	68720	$1x10^{13}$	250/depth	600, 500
wS1 L5 ChR2 stimulation	Rbp4-cre	pAAV-hSynapsin-FLEX-soCoChR-GFP	107712	$1.0x10^{13}$	500	500
wMC ChR2 stimulation	C57BL/6	pAAV-CaMKIIa-hChR2(H134R)-mCherry	26975	$2.30x10^{13}$	100/depth	800, 600, 400, 200
wMC NpHR inhibition	C57BL/6	pAAV-CaMKIIa-eNpHR 3.0-EYFP	26971	$1.0x10^{13}$	350/depth	600, 300

Intrinsic optical imaging

1-2 days before recordings, mice were anesthetized with 3 to 4% isoflurane, and placed under a CMOS camera (Blackfly USB3, Teledyne FLIR) equipped with a DLSR macrolens (Nikon). If necessary, we further thinned the skull region. The camera and lens were angled for a perpendicular visualization of the barrel cortex. We maintained a light anesthesia for the animal (~0.75% isoflurane) and a 2X higher oxygen flow rate than our cranial window surgical procedures (~0.2 L/min) to maintain relatively high blood oxygenation. An infrared heating pad was used to maintain the body temperature during anesthesia. We filled the skull area with mineral oil and used a coverslip to planarize the surface for a stable image intensity. The thinned, soaked bone was transparent to the point of easily visualizing the underlying vasculature. We used a multi-wavelength LED for all imaging (Lumencor, Spectral X). We trimmed all whiskers other than the C row and placed a small metal hook over the C1 whisker. The hook was attached to a stepper motor (NEMA 17 motor, DM556 motor driver) controlled by an Arduino UNO for rapid vibration at 30 Hz. An image of the vasculature was taken as a reference with green LED illumination. A NIR LED was used to illuminate the thinned scalp for intrinsic imaging. Custom-written MATLAB (MathWorks) and Arduino codes were used for controlling the experiment. During each imaging trial, we recorded 100 frames of baseline data (25 fps) followed by an additional 100 frames during whisker stimulation. The frames taken before and during whisker deflection were averaged separately and the difference between the two averaged images formed the intrinsic optical signal for one trial. The trial was repeated for 6 to 15 times, and the resultant image from each trial was averaged until the contrast of the image was clear and the barrel distinguishable from the background. Using ImageJ, we smoothed the final image with a gaussian filter and overlaid the intrinsic signal with the vasculature reference.

Passive whisker stimulation:

Awake, head-fixed animals were allowed to run on a wheel but were largely stationary due to the lack of incentive for locomotion (**Figure S2F**). All whiskers were trimmed except for C1. For recording touch-

evoked potentials, we performed passive stimulation of the C1 whisker with a metal pole driven by a motor that fully brushed past the whisker rapidly (NEMA 17 motor, DM556 motor driver) once during a trial. Whiskers were not contacted again, and mice freely whisked after stimulus presentation. We used a trial-to-trial interval of 10-20 s. All experimental systems were synchronized with an Arduino microcontroller (Arduino UNO).

Active-touch: Lick detection

During training the headplate of the mouse was connected to 5V of the Arduino board, and an analog input pin (with a pull-down resistor to ground) connected to the water spout was used to read changes in voltage when the mouse licked. During electrophysiology, to avoid lick induced artifacts in the recording quality, the analog signal from a piezoelectric bender (BA3502, Micromechatronics, Inc.) attached to the lick port was used as lick signal. This signal was amplified by an operational amplifier and was sent in real time to an Arduino microcontroller and Intan 512 controller. The signal was synchronized with neural data via external triggers generated by the National Instruments card. Lick signal was considered a lick if it crossed a threshold of 2 volts.

Two-photon imaging

Two-photon imaging was performed using a laser-scanning microscope (Sutter MOM, Sutter Instruments) fitted with resonant scanning mirrors (Vidrio Tech). We used a tunable femtosecond pulsed laser (Insight X3, Spectra Physics) for the excitation source with beam intensity controlled with an electro-optical modulator (Conoptics, model 350-50). We used a Nikon 16X/0.8NA water-immersion objective with a 3 mm working distance and imaged at wavelengths of 940 nm with powers of <55 mW at the focal plane. All experiments were controlled with ScanImage software with a 512x512 pixel field of view sampled at 30 Hz. To locate the grid on the brain surface without damaging the metal or parylene, we used low powers (<5 mW). We set the z = 0 focal plane to the grid center, then lowered the objective to the depth of interest before increasing the laser power. For L2/3 imaging, we performed touch trials at multiple depths between 150-275 um at 1.5X magnification with 30-50 trials per depth and a minimum spacing between focal planes of 25 µm. For dendritic imaging, we used a 3-5X magnification and imaged at depths between 50 µm (i.e., tufts) and 200 µm (apicals).

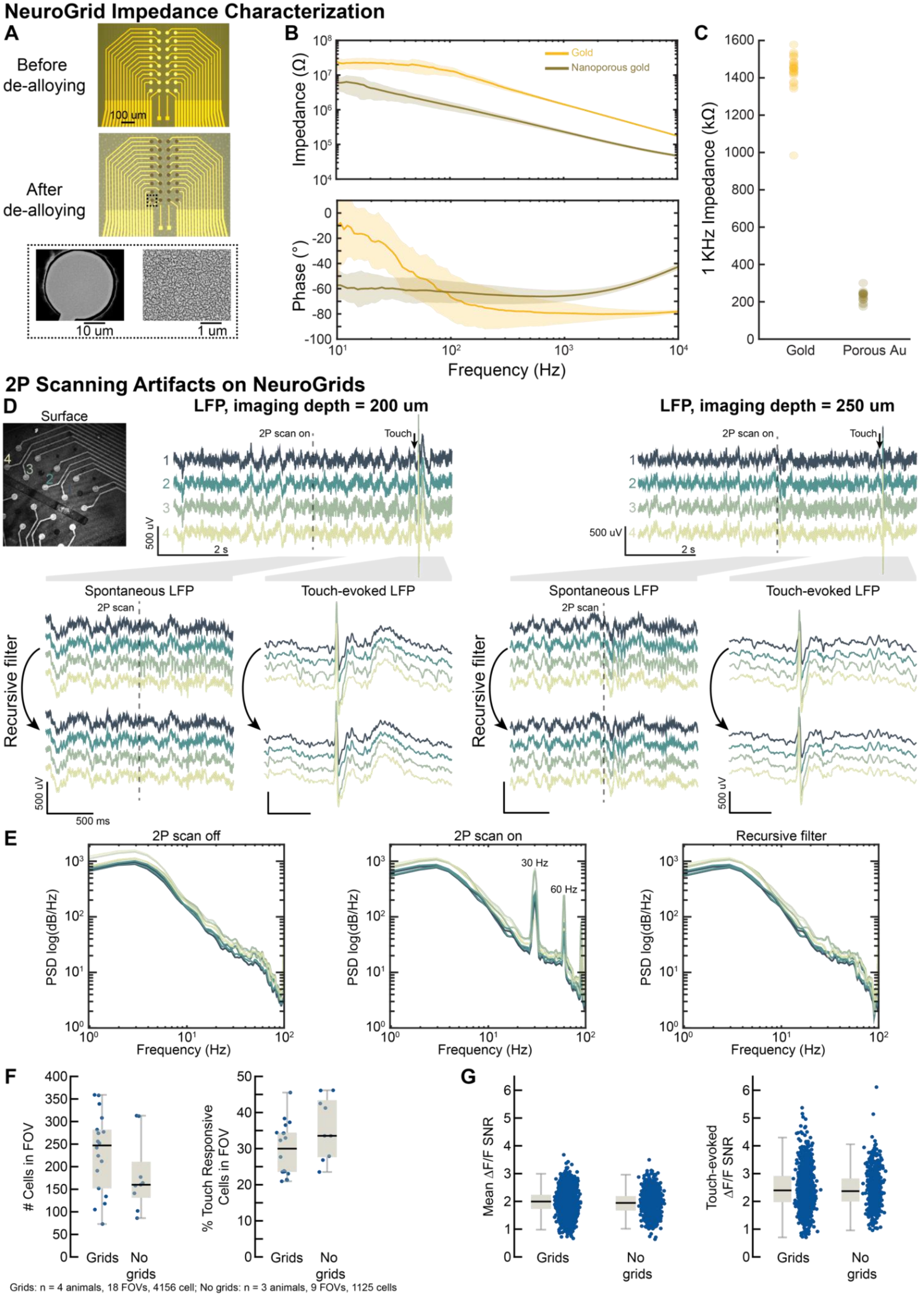
NeuroGrid Impedance Characterization
A
Before de-alloying
100 um
After de-alloying
10 um
1 um
B
Gold
Nanoporous gold
Impedance (Ω)
Phase (°)
Frequency (Hz)
C
1 KHz Impedance (kΩ)
Gold
Porous Au
2P Scanning Artifacts on NeuroGrids
D
Surface
LFP, imaging depth = 200 um
LFP, imaging depth = 250 um
2P scan on
Touch
500 uV
2 s
Spontaneous LFP
Touch-evoked LFP
2P scan
Recursive filter
500 ms
E
2P scan off
2P scan on
Recursive filter
30 Hz
60 Hz
PSD log(dB/Hz)
Frequency (Hz)
F
Cells in FOV
% Touch Responsive Cells in FOV
Grids
No grids
G
Mean ΔF/F SNR
Touch-evoked ΔF/F SNR
Grids: n = 4 animals, 18 FOVs, 4156 cell; No grids: n = 3 animals, 9 FOVs, 1125 cells

Figure S1

Figure S1. NeuroGrid electrochemical impedance and imaging characterization.

(A) Optical micrographs of an on-chip NeuroGrid before and after the de-alloying process in nitric acid. Insets are scanning-electron micrographs of one recording pad, which shows the porosity.

(B) Impedance and phase comparison of recording sites composed of only gold and gold+nanoporous gold.

(C) 1 KHz impedance comparison of gold vs nanoporous gold recording sites.

(D) (Top) Micrograph shows NeuroGrid orientation on the brain surface. Selected channels are labeled. LFP traces show recordings from selected recording sites at imaging depths of 200 and 250 µm. Two-photon laser scanning turns on halfway through the traces (dashed line), and a touch occurs near the end. (Bottom) LFP traces when the two-photon scanning turns on and when touch occurs. The arrow indicates the same traces after applying a recursive notch filter to remove scanning noise. Scale bars: 500 µV and 500 ms.

(E) Power spectrum during periods with no imaging (left), during two-photon imaging (center), and two-photon imaging data after the recursive filter is applied (right). Power spectrums were calculated during spontaneous LFP activity.

(F) Total number of cells and touch-responsive cells in wS1 in the FOV with and without simultaneous surface grid recordings.

(G) Comparison of the ΔF/F SNR for all calcium events (left) and touch-evoked calcium events (right) during recordings with and without the NeuroGrid.

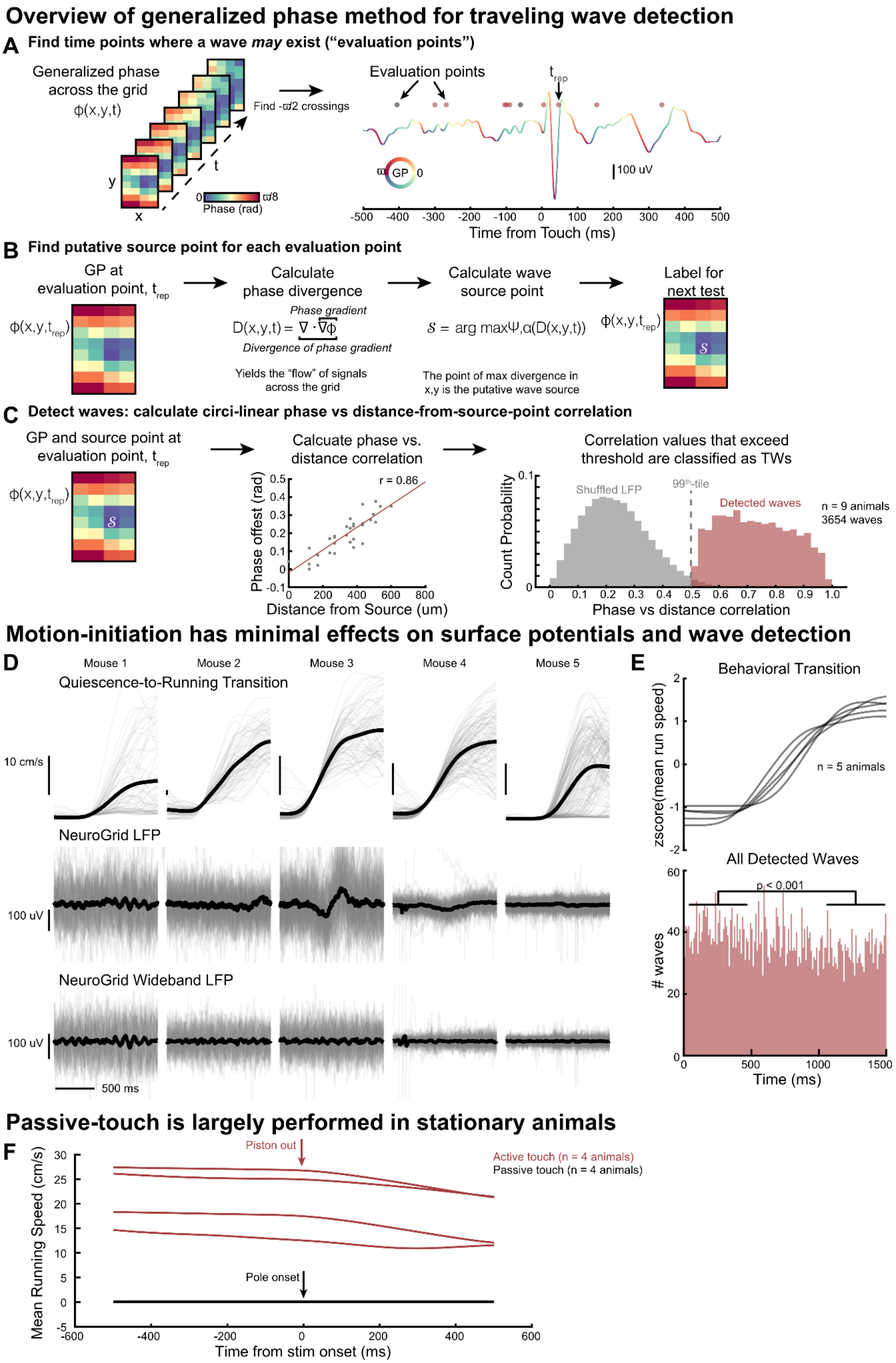

Overview of generalized phase method for traveling wave detection
A Find time points where a wave may exist ("evaluation points")
Generalized phase across the grid
ϕ(x,y,t)
Find -ϖ/2 crossings
y
x
t
0
ϖ/8
Phase (rad)
Evaluation points
t rep
ϖ GP 0
100 uV
-500 -400 -300 -200 -100 0 100 200 300 400 500
Time from Touch (ms)
B Find putative source point for each evaluation point
GP at evaluation point, t rep
ϕ(x,y,t rep)
Calculate phase divergence
Phase gradient
D(x,y,t) = ∇ · ∇ϕ
Divergence of phase gradient
Yields the "flow" of signals across the grid
Calculate wave source point
S = arg maxΨ,α(D(x,y,t))
The point of max divergence in x,y is the putative wave source
Label for next test
ϕ(x,y,t rep)
S
C Detect waves: calculate circi-linear phase vs distance-from-source-point correlation
GP and source point at evaluation point, t rep
ϕ(x,y,t rep)
S
Calcuate phase vs. distance correlation
r = 0.86
Phase offest (rad)
Distance from Source (um)
Correlation values that exceed threshold are classified as TWs
Count Probability
Shuffled LFP
99th-tile
Detected waves
n = 9 animals
3654 waves
Phase vs distance correlation
Motion-initiation has minimal effects on surface potentials and wave detection
D
Mouse 1
Mouse 2
Mouse 3
Mouse 4
Mouse 5
Quiescence-to-Running Transition
10 cm/s
NeuroGrid LFP
100 uV
NeuroGrid Wideband LFP
100 uV
500 ms
E
Behavioral Transition
zscore(mean run speed)
n = 5 animals
All Detected Waves
p < 0.001
waves
Time (ms)
Passive-touch is largely performed in stationary animals
F
Piston out
Active touch (n = 4 animals)
Passive touch (n = 4 animals)
Pole onset
Mean Running Speed (cm/s)
Time from stim onset (ms)

Figure S2

Figure S2. Overview of generalized phase pipeline and locomotion effects on NeuroGrid LFP

(A) Workflow for finding points-of-interest for potential TW detection. (Left) Representative time points of generalized phase across the grid calculated from the LFP. (Right) We detected all $-\pi/2$ phase crossings in the touch trial and label these as “evaluation points” for more testing to determine if a TW exists.

(B) For each evaluation point, we determined the putative source point by calculating the divergence of the phase gradient across the grid.

(C) For each evaluation point, we then calculated the circular-linear phase vs. distance-from-source-point correlation. Points with strong correlation values were then determined to be TWs. Significant correlation values were found by first constructing a “null distribution” of correlation values for each animal. We did this by shuffling the LFP location across the grid, running GP analysis, and calculating the correlation value for all pseudo-evaluation points (50 shuffle iterations were performed for each trial for each animal). The 99th percentile of the null distribution was used as a threshold for traveling wave detection in unshuffled data. In panel (A), black evaluation points had correlation values below the threshold.

(D) We considered whether the late wave could be confounded by behavioral state transitions. In the absence of touch, we analyzed animal locomotion and detected spontaneous behavioral transitions from quiescence to running. For these behavioral transitions, we aligned the full LFP spectrum (middle traces) and wideband filtered LFP used for traveling wave analysis (bottom traces). We observed no consistent influence of behavioral state transitions on the NeuroGrid LFP.

(E) For the behavioral transitions in the absence of touch, we also performed traveling wave detection. We detected spontaneous waves in the LFP in a 500 ms period prior to running and 500 ms window after running onset and found a reduced number of waves during running (bootstrap followed by a signed-rank Wilcoxon test). However, the behavioral transition does not appear to initiate waves as in the case of whisker touch.

(F) Average running speed across all animal trials during the active and passive touch paradigms.

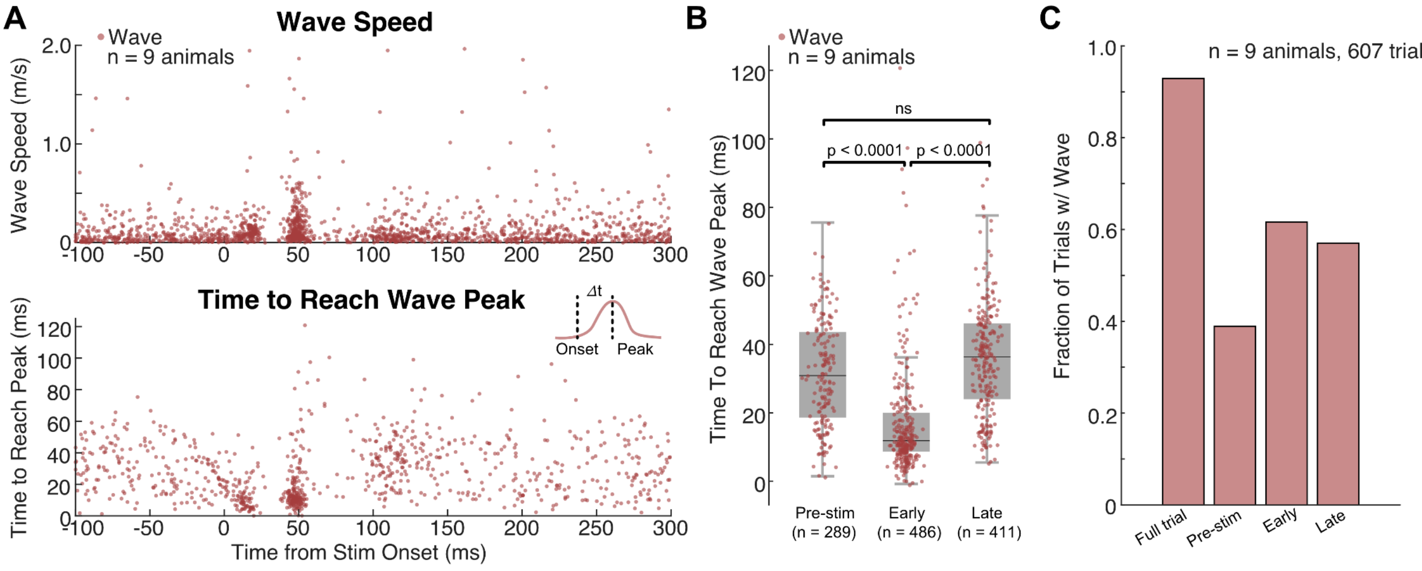

Figure S3. Additional wave metrics during passive touch.

(A) (Top) Raster plot of wave speed as a function of the wave onset throughout touch trial. (Bottom) Raster plot of the time for a wave to reach its peak as a function of wave onset throughout each trial (n = 9 animals, 1639 total waves)

(B) Further quantification of the time for waves to reach their peak based on occurrence during the pre-stimulation period, early period, or late period. (n = 9 animals, 289 pre-stimulus waves, 486 early waves, 411 late waves; ns = not significant, $p < 0.001$ Kruskal-Wallis with a post-hoc Dunn-Sidak test).

(C) Fraction of trials with a wave during the entire trial, pre-stimulation period, early period, or late period.

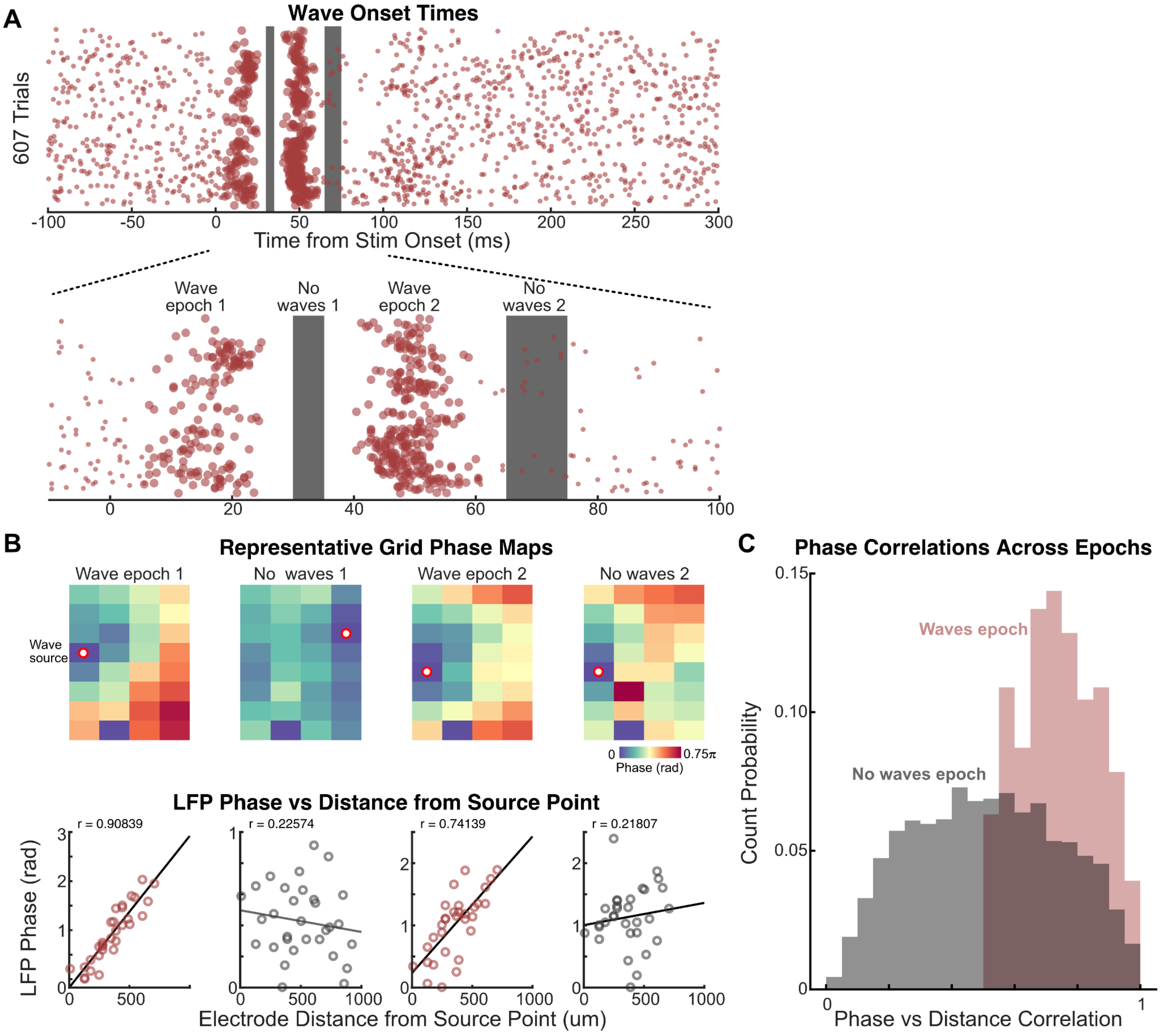

Figure S4. Phase distributions across the grid during wave and no-wave epochs after touch.

(A) Replot of Figure 2D showing detected traveling wave times across animals. Inset focuses on the first 100 ms after touch, where clear "wave" and "no-wave" epochs emerge.

(B) We detected pseudo-waves during the "no-waves" epoch and compared these to true detected waves during each "wave" epoch. (Top) Phase maps of representative waves and no-waves. (Bottom) Phase distribution across the grid organized by distance from the determined source point. The linear fits provide an indication of whether the phase distribution meets the criteria for classification as a true wave (see Figure S2C).

(C) Phase vs distance correlation values (i.e. linear fits from (C)) for all wave and no-wave epochs.

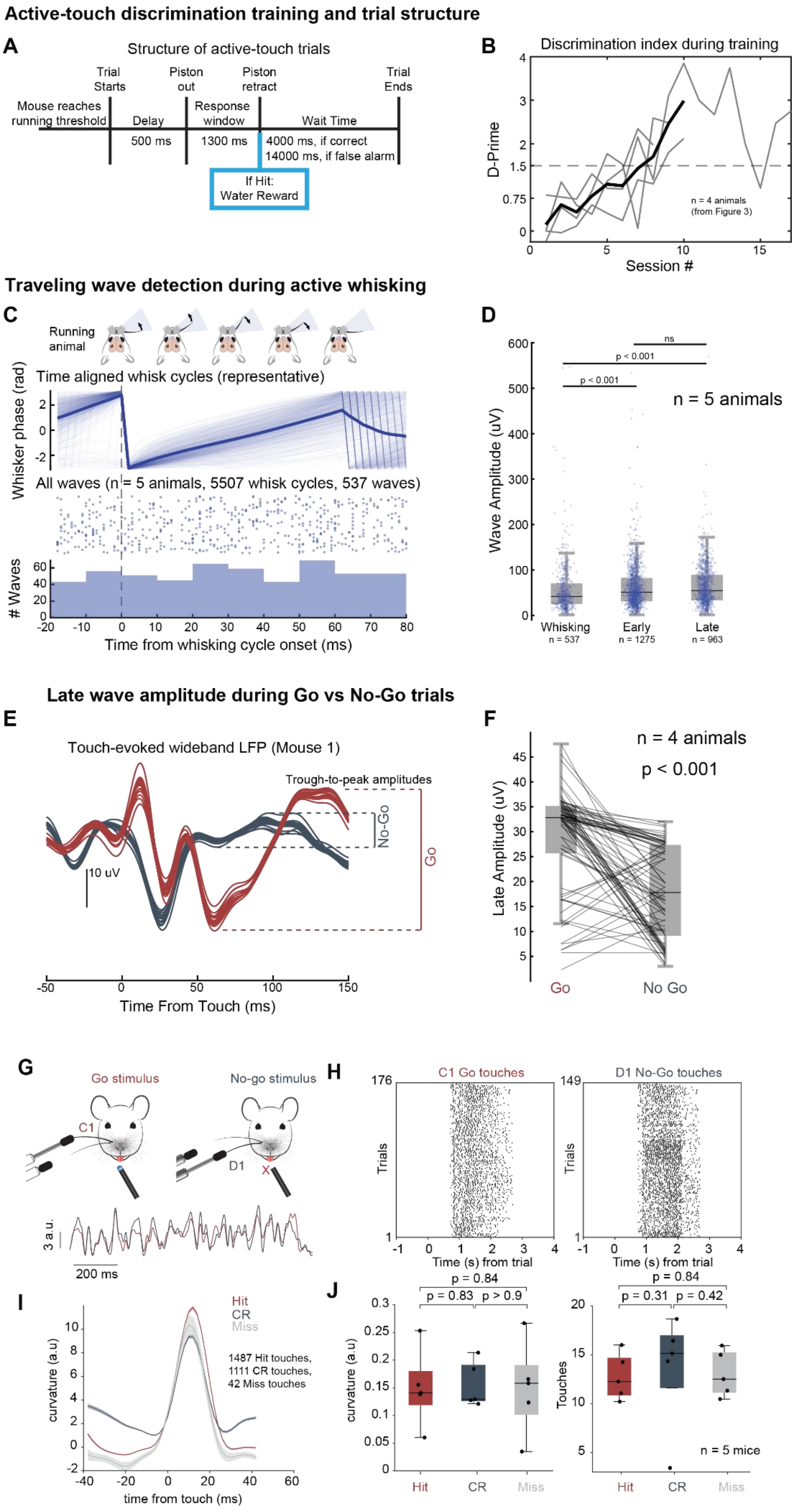

Active-touch discrimination training and trial structure
A
Structure of active-touch trials
Trial Starts
Piston out
Piston retract
Trial Ends
Mouse reaches running threshold
Delay
500 ms
Response window
1300 ms
Wait Time
4000 ms, if correct
14000 ms, if false alarm
If Hit: Water Reward
B
Discrimination index during training
D-Prime
Session #
n = 4 animals (from Figure 3)
Traveling wave detection during active whisking
C
Running animal
Time aligned whisk cycles (representative)
Whisker phase (rad)
All waves (n = 5 animals, 5507 whisk cycles, 537 waves)
Waves
Time from whisking cycle onset (ms)
D
Wave Amplitude (uV)
n = 5 animals
p < 0.001
p < 0.001
ns
Whisking
n = 537
Early
n = 1275
Late
n = 963
Late wave amplitude during Go vs No-Go trials
E
Touch-evoked wideband LFP (Mouse 1)
Trough-to-peak amplitudes
No-Go
Go
10 uV
Time From Touch (ms)
F
n = 4 animals
p < 0.001
Late Amplitude (uV)
Go
No Go
G
Go stimulus
No-go stimulus
C1
D1
3 a.u.
200 ms
H
C1 Go touches
D1 No-Go touches
Trials
Time (s) from trial
I
Hit
CR
Miss
1487 Hit touches,
1111 CR touches,
42 Miss touches
curvature (a.u)
time from touch (ms)
J
p = 0.84
p = 0.83
p > 0.9
p = 0.31
p = 0.42
curvature (a.u)
Touches
n = 5 mice
Hit
CR
Miss

Figure S5.

Figure S5. Overview of two-whisker, active-touch discrimination behavior.

(A) Details of the single-trial structures conducted during the active-touch paradigm.

(B) Discrimination index for the two-whisker operant behavior during training sessions. We considered a discrimination index of 1.5 as expert behavior.

(C) We considered that whisking itself may evoke wS1 traveling waves. We detected waves during free whisking with no touch-related objects to test this. (Top) Whisking phase. The schematic shows different whisker positions and their relationship to the whisking phase. The plot shows time-aligned whisking phases for a representative animal. We limited our analyses to whisking cycles that lasted a minimum of 60 ms. (Middle) Raster plot of all detected traveling wave onsets in the wideband LFP across all animals. (Bottom) Histogram of wave onset times. There is no clear correlation between the whisking phase and traveling wave onset (n = 5 animals, 5507 total whisking cycles, 537 total detected waves).

(D) Traveling wave amplitude for free whisking, touch-evoked early waves (0-50 ms post touch), and touch-evoked late waves (50-100 ms post touch). Touch-evoked amplitudes are significantly stronger (n = 5 animals, 537 whisking-only waves, 1275 early waves, 963 late waves, p-values determined with a Kruskal-Wallis test with a *post-hoc* Dunn-Sidak test).

(E) Representative active-touch evoked NeuroGrid LFP for Go (C1 touch) and No-Go (D1 touch) trials. The arrow indicates the trough-to-peak region of the late wave that was quantified.

(F) The late wave trough-to-peak amplitude quantified across all electrodes was significantly higher for Go trials, indicating reward reinforcement enhances the late wave (n = 4 animals, p-value determined with a signed-rank Wilcoxon test).

(G) (Top) Schematic of active behavior paradigm involving two-whisker discrimination. (Bottom) Trial average whisking response for the representative animal.

(H) A representative touch raster across one recording session for Go and No-Go trials.

(I) Whisker variance between Hit and Correct reject trials (n = 325 trials).

(J) (Left) No difference was observed in the variation of whisker movement across trials and between task conditions (rank-sum Wilcoxon test, n = 5 mice). (Right) No difference was observed in the number of sampled touches between task conditions (rank-sum Wilcoxon test, n = 5 mice)

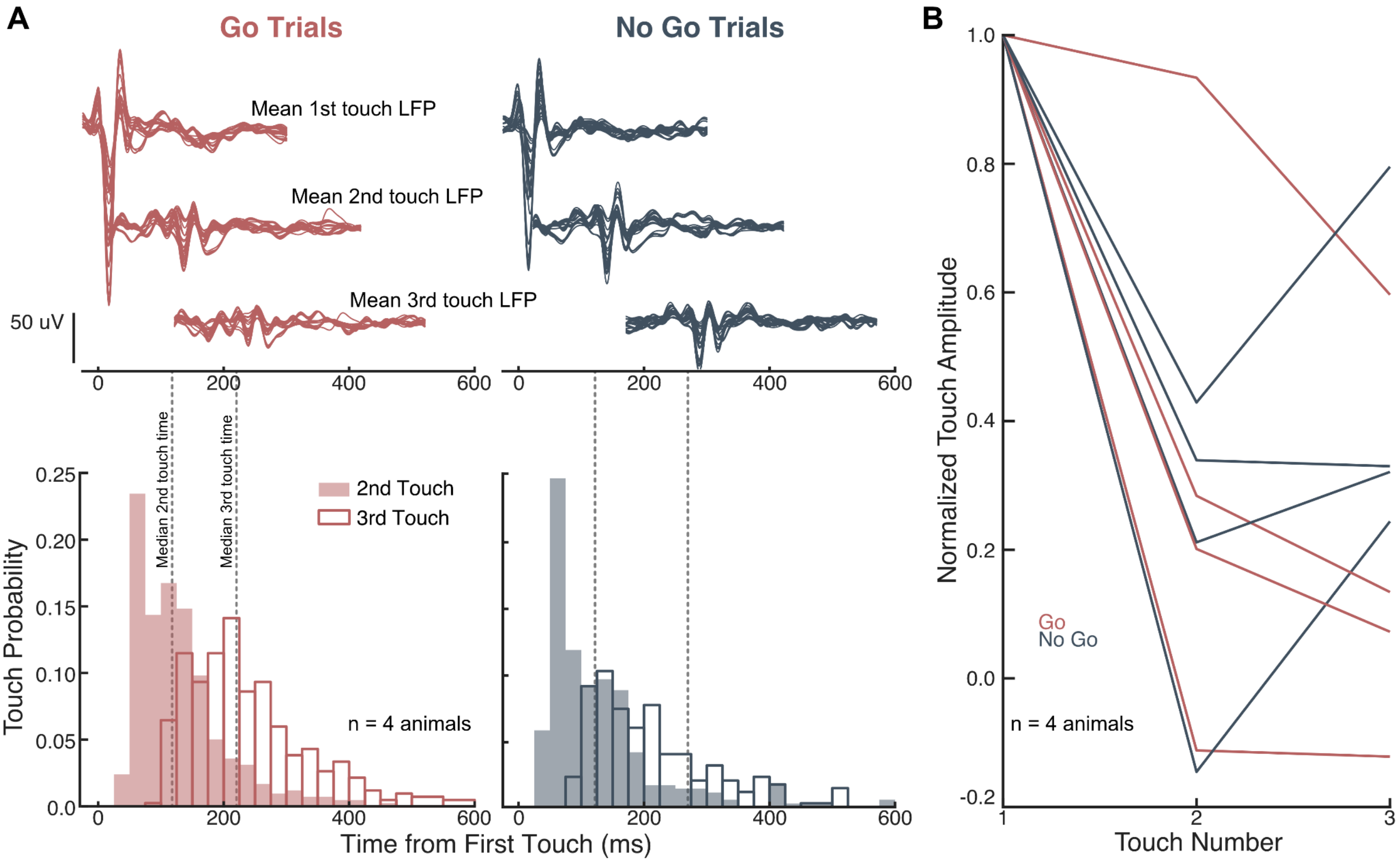

Figure S6

Figure S6. Consecutive touches reduce evoked LFP

(A) (Top) Mean LFP for a representative animal for the first, second, and third consecutive touches occurring within Go and No-Go trials. Mean LFP is for all detected touches, not those separated by a 100 ms interval as shown in the main figure. For wave detection and Figure 3, only touches with an interval >100 ms were used. (Bottom) Histogram of touch times for all combined animals (n = 4) for the second and third touches in Go and No-Go trials. Dashed lines indicate the median touch times. Histogram

(B) Average LFP amplitude across the grid normalized to the first touch. Each line indicates data from a single animal for Go and No-Go trials.

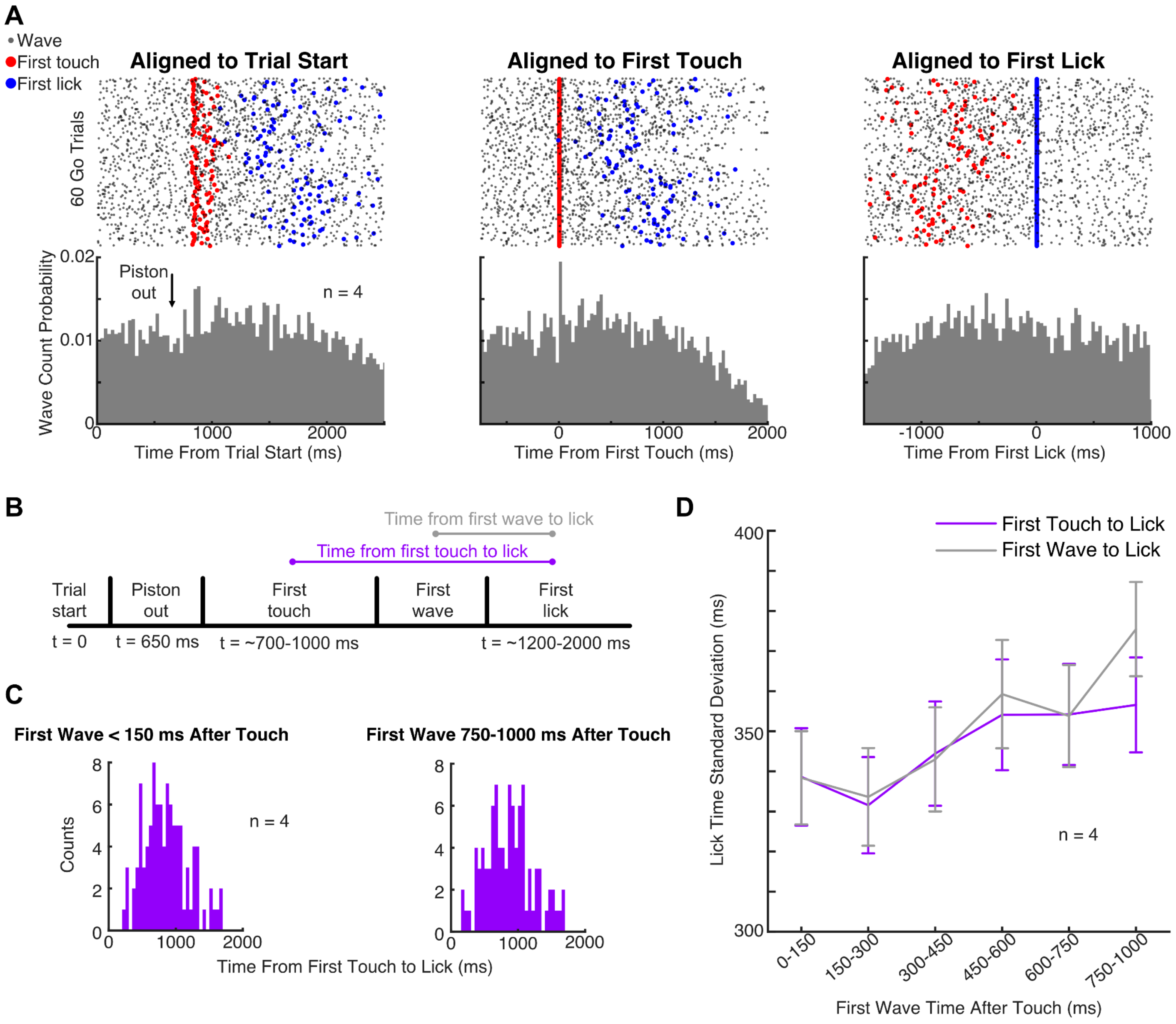

Figure S7. Wave and lick times during active touch Go trials

(A) Distribution of wave onset times during active touch Go trials with respect to the trial onset, the first touch time, and the first lick time. Top raster plot is for a representative animal. Bottom histogram is combined data across all animals.

(B) Schematic of a Go trial with approximate timeline of events.

(C) Distribution of lick times following the first touch. Left plot shows lick times when the first wave occurred within 150 ms of the first touch. Right plot shows lick times when the first wave occurred 750-1000 ms after the first touch (i.e. the first and initial touches did not evoke a detectable wave).

(D) Standard deviation of lick times (i.e. plots in C). Trials that evoke a wave within 150 ms of the first touch show a slight sharpening of lick output times.

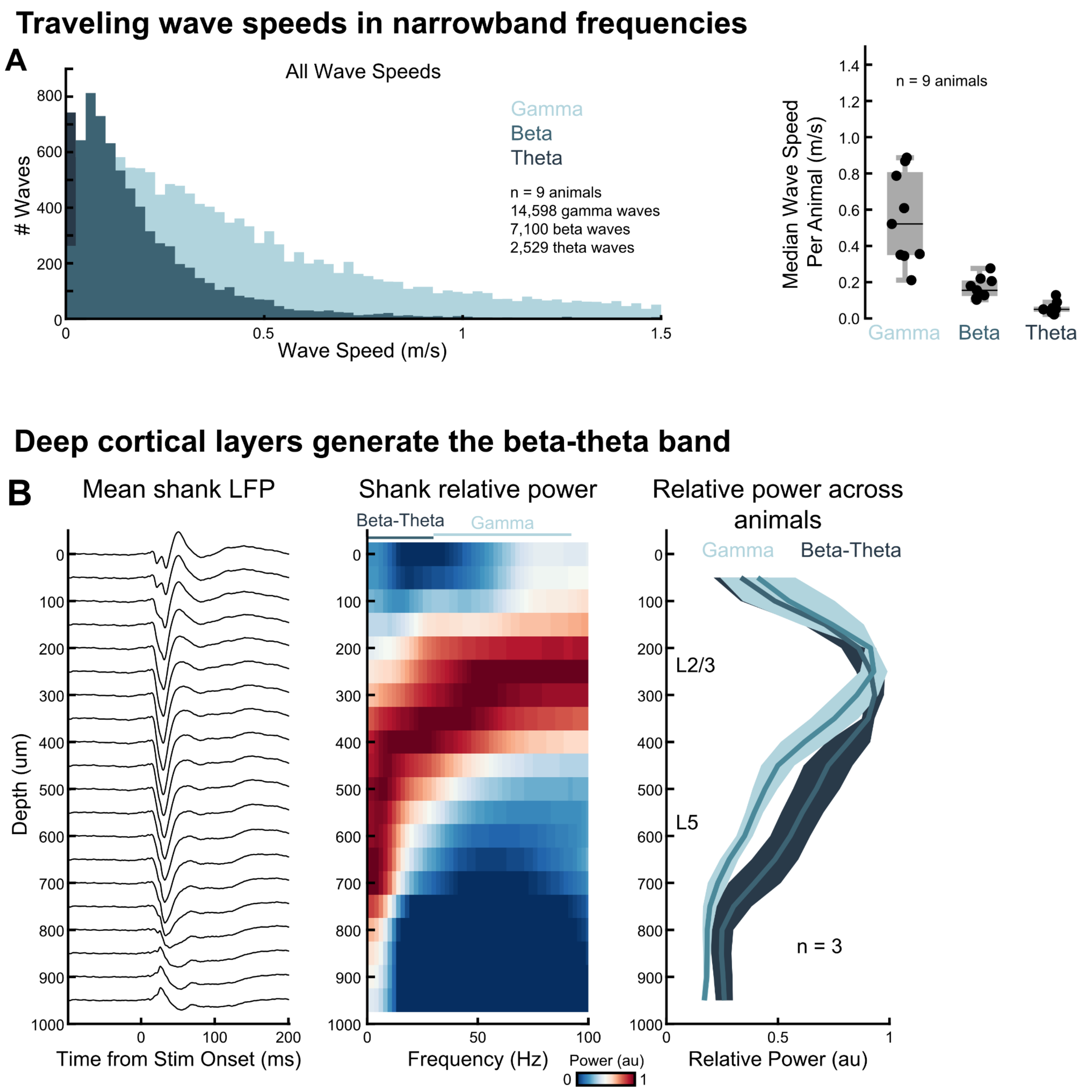

Figure S8.

Figure S8. Narrowband frequency analysis of wS1 traveling waves with NeuroGrids and silicon probes.

(A) (Left) Speeds for all detected traveling waves on the NeuroGrid in wS1 in the gamma, beta, and theta bands (n = 9 animals, 14,589 gamma waves, 7,100 beta waves, 2,529 theta waves). (Right) Median wave speed per animal for each frequency band.

(B) (Left) Mean touch-evoked LFP on a wS1 silicon probe for a representative animal. (Center) Representative relative power in each frequency band as a function of cortical depth. This laminar profile shows that the infragranular layers (>400 µm) have enhanced beta-theta power (4-30 Hz), and the supragranular layers are dominated by the gamma band (30-90 Hz). (Right) Relative laminar power for the beta-theta and gamma bands across animals (n = 3). Again, the infragranular layers—and most notably L5—are primarily driven by the beta-theta band. Shaded error is the standard error of the mean.

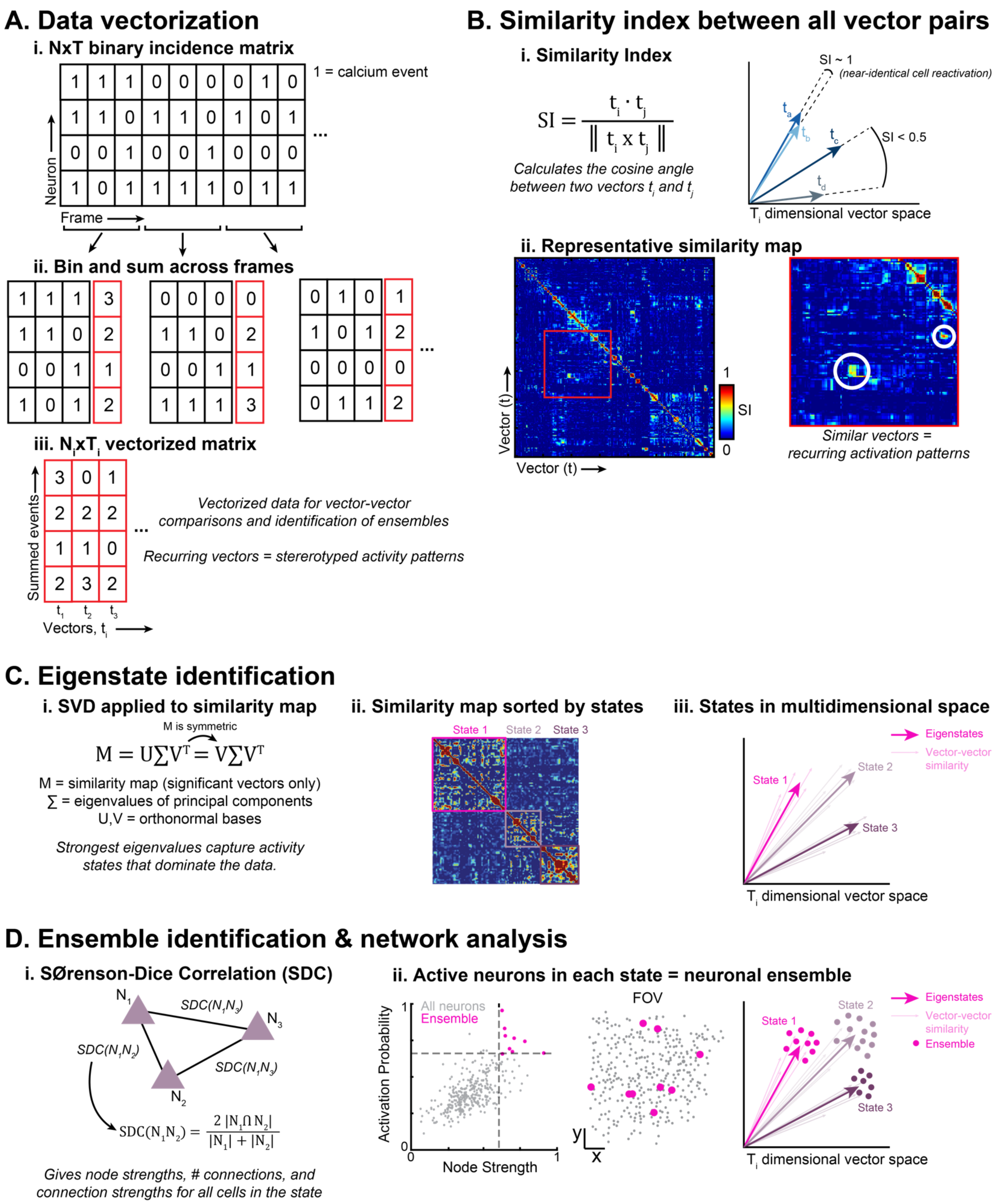
A. Data vectorization
i. NxT binary incidence matrix
1 = calcium event
Neuron
Frame
ii. Bin and sum across frames
iii. $N_i x T_i$ vectorized matrix
Summed events
Vectors, t_i
Vectorized data for vector-vector comparisons and identification of ensembles
Recurring vectors = stererotyped activity patterns
B. Similarity index between all vector pairs
i. Similarity Index
$SI = \frac{t_i \cdot t_j}{\| t_i \, x \, t_j \|}$
Calculates the cosine angle between two vectors t_i and t_j
SI ~ 1
(near-identical cell reactivation)
SI < 0.5
T_i dimensional vector space
ii. Representative similarity map
Vector (t)
SI
Similar vectors = recurring activation patterns
C. Eigenstate identification
i. SVD applied to similarity map
M is symmetric
$M = U\sum V^T = V\sum V^T$
M = similarity map (significant vectors only)
∑ = eigenvalues of principal components
U,V = orthonormal bases
Strongest eigenvalues capture activity states that dominate the data.
ii. Similarity map sorted by states
State 1
State 2
State 3
iii. States in multidimensional space
Eigenstates
Vector-vector similarity
T_i dimensional vector space
D. Ensemble identification & network analysis
i. SØrenson-Dice Correlation (SDC)
$SDC(N_1N_3)$
$SDC(N_1N_2)$
$SDC(N_1N_3)$
$SDC(N_1N_2) = \frac{2\,|N_1 \cap N_2|}{|N_1| + |N_2|}$
Gives node strengths, # connections, and connection strengths for all cells in the state
ii. Active neurons in each state = neuronal ensemble
All neurons
Ensemble
Activation Probability
Node Strength
FOV
Eigenstates
Vector-vector similarity
Ensemble
T_i dimensional vector space

Figure S9

Figure S9. Ensemble detection and network connectivity analysis pipeline.

(A) Calcium event binning and vectorization procedure. With this technique, we can use vector-based calculations to determine common patterns of reactivation across the cortical landscape.

(B) Dot-product vector multiplication for similarity index calculation. This allows for comparing all vectors in the data set. Vectors with a high similarity will have nearly identical cellular activation patterns.

(C) SVD procedure for identifying activity states that dominate the calcium data.

(D) SDC calculations provide detailed network connectivity metrics and the cells that primarily compose the eigenstates.

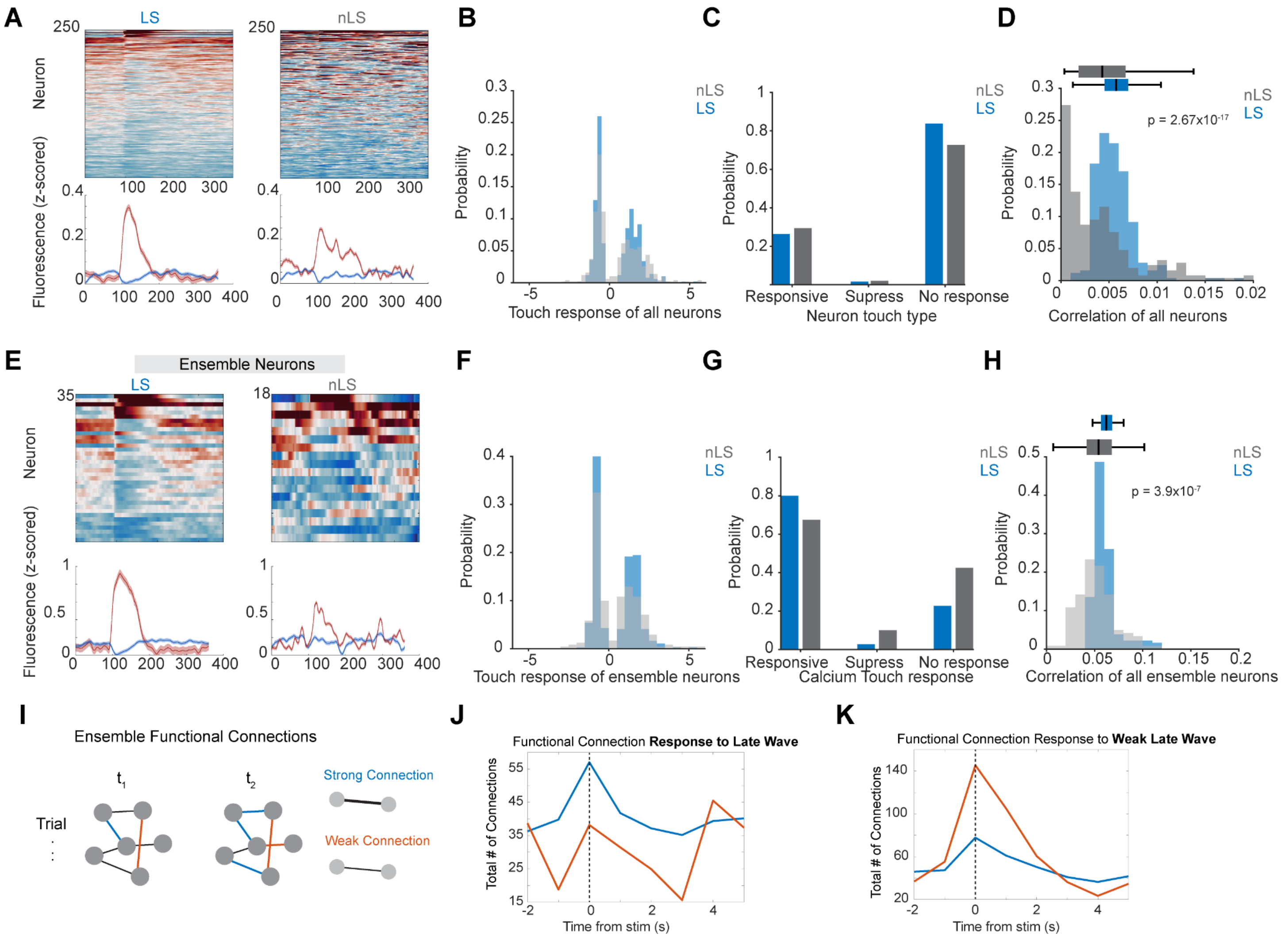

Figure S10. Additional analysis of population-wide and ensemble neurons during strong and weak late wave trials.

(A) Trial averaged the calcium response of all neurons in an example field of view during strong late wave (LS) and weak late wave trials (nLS) (top, n = 251 neurons across 45 trials). (Bottom) Trial averaged fluorescence response across FOV (n = 10).

(B) Touch responsivity of neurons across late wave and weak late wave trials (n = 540 neurons across 10 FOV and 3 mice).

(C) Percentage of total neurons that are statistically responsive, suppressive, or not responsive.

(D) Population pairwise correlation across late wave and weak late wave trials (Wilcoxon rank-sum, $p<0.001$)

(E-F) Same analysis as (A-D) but across detected ensemble neurons.

(I-K) Number of weak and strong functional connections in ensembles as a function of time (1 second binned analysis) during touch-evoked trials with a strong and weak late wave LFP. Note differences in total number of functional connections per time point.

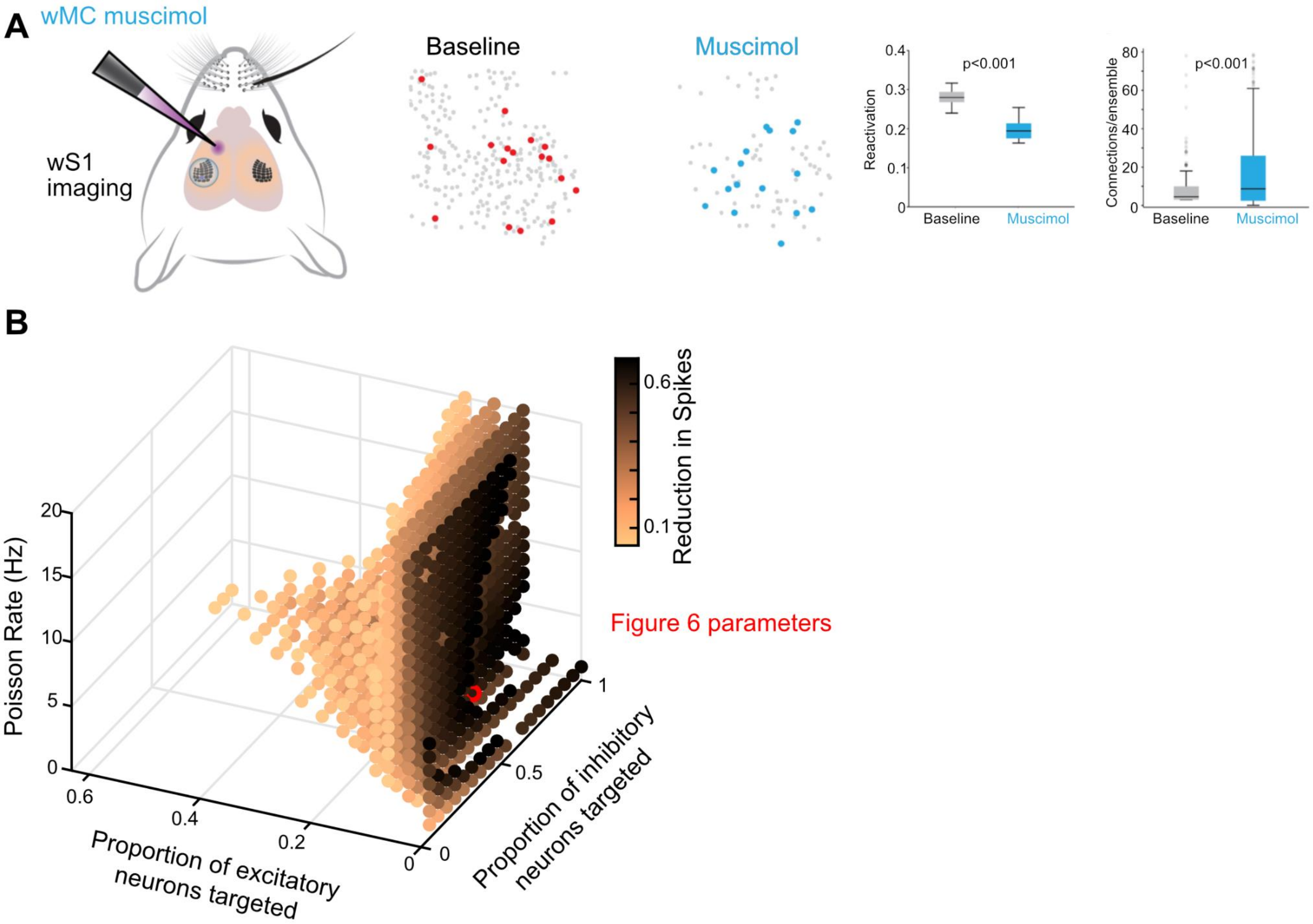

Figure S11.

Figure S11. Extended computational and experimental results on feedback modulation of sensory cortex.

(A) Muscimol inhibition of wMC during wS1 imaging and passive whisker stimulation. We observed decreased ensemble reactivation and increased functional connectivity during wMC silencing, indicating reduced ensemble stability, reduced sparse representations, and opposing the effects during strong late waves (see Figure 5). (n = 4 animals, $p < 0.001$, ranked-sum Wilcoxon test).

(B) Feedback increases the sparsity of traveling waves across a broad range of ratios and firing rates. Plotted are results from simulations of the sparse-wave network, with different proportions of excitatory neurons in the patch targeted by feedback projections, proportion of inhibitory neurons targeted, and Poisson rate for feedback. Each dot plotted in the figure represents a simulation where sparsity was increased by feedback by approximately 75% or higher (measured as the percentage decrease in spiking activity caused by the feedback compared to the without-feedback control). These results demonstrate that, for a broad range of values, feedback that predominantly targets inhibitory neurons can substantially increase the sparsity of waves traveling over the spiking network model.

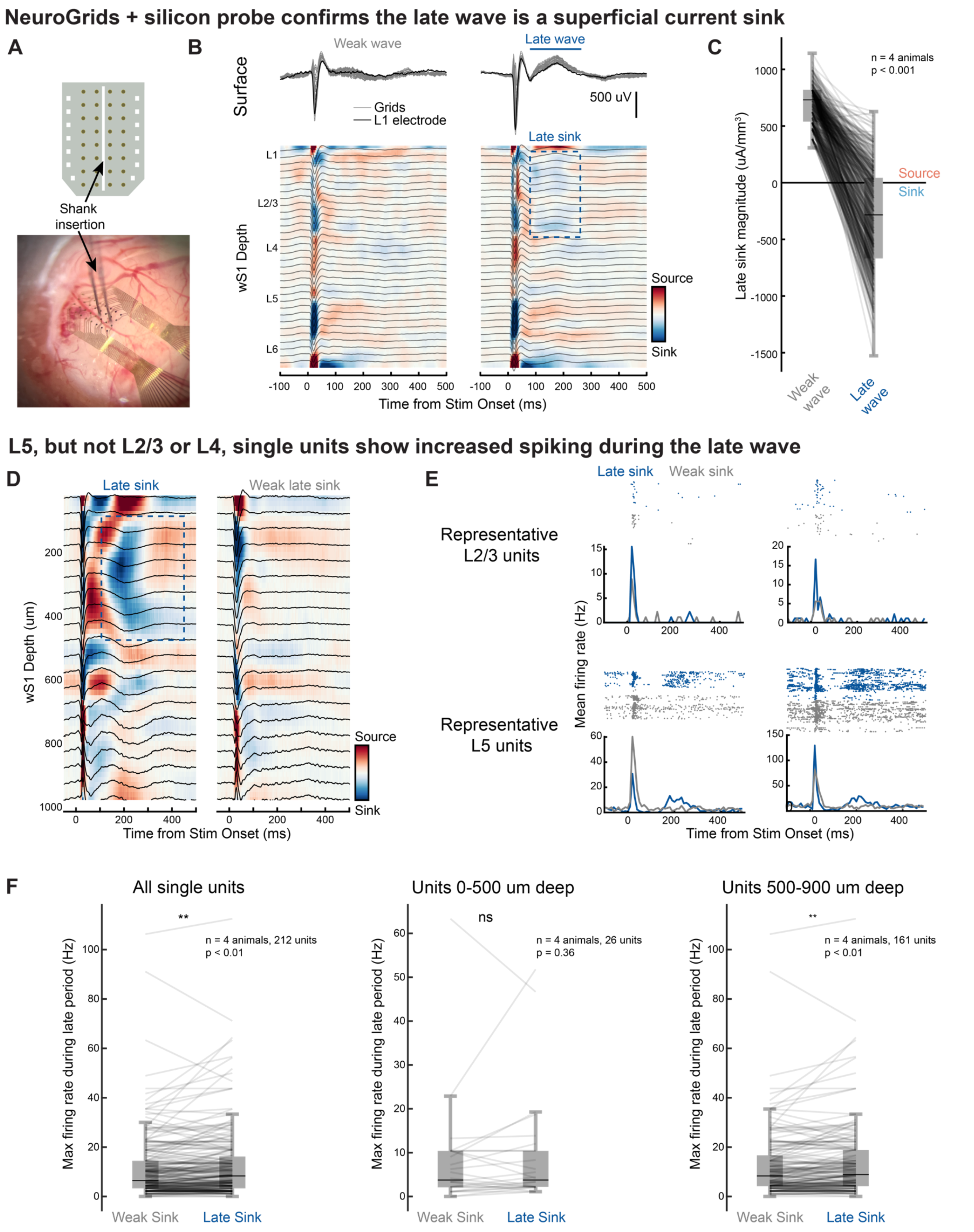
NeuroGrids + silicon probe confirms the late wave is a superficial current sink
A
B
C
Shank insertion
Surface
Weak wave
Late wave
Grids
L1 electrode
500 uV
Late sink
L1
L2/3
L4
L5
L6
wS1 Depth
Source
Sink
-100 0 100 200 300 400 500
Time from Stim Onset (ms)
n = 4 animals
p < 0.001
Late sink magnitude (uA/mm³)
1000
500
0
-500
-1000
-1500
Weak wave
Late wave
L5, but not L2/3 or L4, single units show increased spiking during the late wave
D
E
Late sink
Weak late sink
wS1 Depth (um)
200
400
600
800
1000
Time from Stim Onset (ms)
Weak sink
Representative L2/3 units
Representative L5 units
Mean firing rate (Hz)
F
All single units
Units 0-500 um deep
Units 500-900 um deep
**
ns
n = 4 animals, 212 units
p < 0.01
n = 4 animals, 26 units
p = 0.36
n = 4 animals, 161 units
p < 0.01
Max firing rate during late period (Hz)
Weak Sink
Late Sink

Figure S12.

Figure S12. The late wave is a supragranular current sink with non-somatic origins.

(A) Schematic and optical micrograph demonstrating how thin silicon probes are inserted through a small through-hole in the NeuroGrid.

(B) Representative simultaneous surface and depth recording. We sorted NeuroGrid trials based on the strength of the late wave and performed CSD analyses on the corresponding laminar recordings. (Top) Average LFP across all NeuroGrid channels for late-wave and weak-late-wave trials (gray). Also shown is the mean LFP for the superficial shank electrode (black). (Bottom) Average shank LFP for each group and the corresponding CSD from the average waveforms. The late wave correlates with a clear, delayed L2/3 current sink.

(C) Average late sink magnitude during late-wave and weak-late-wave trials. The late sink strengthens with a strong late wave. ($n = 4$ animals, p-value determined with a signed-rank Wilcoxon test following bootstrapping of LFP single trials).

(D-F) Silicon probe recordings with no surface grids for single-unit analyses during the late sink.

(D) Mean touch-evoked LFP across the laminar probe for a representative animal and corresponding CSD. Trials were sorted by the strength of the late L2/3 sink.

(E) Representative single units in L2/3 (200 and 250 µm depth, respectively) and L5 (600 and 650 µm depth, respectively). Both a raster plot of spike times for individual trials and the average firing rate across trials are shown for each unit. Trials were sorted based on the strength of the L2/3 late sink (strong late sink in blue and weak late sink in gray). Notably, L5 neurons show an additional bump in their firing rates during the late sink period (100-300 ms), while L2/3 neurons do not.

(F) We compared strong- vs. weak-late-sink firing rates across all detected single units (left), supragranular single units (center), and infragranular single units (right) ($n = 4$ animals). Across all neurons, we observed an increase in the maximum firing rate during the late period (100-300 ms) in trials with a prominent late sink (left, $n = 212$ units, $p < 0.01$). This increase was not observed in units with a depth of less than 500 µm (center, 26 units, $p = 0.36$). This effect was restricted to infragranular single units with a depth greater than 500 µm (right, $n = 161$ units, $p < 0.01$). A signed-rank Wilcoxon test was used for all comparisons. These results indicate that the superficial late sink is not driven by an increase in superficial-layer spiking. However, increased spiking in L5 neurons during the late sink suggests that L5 spiking drives apical dendrite electrogenesis and the delayed current sink.

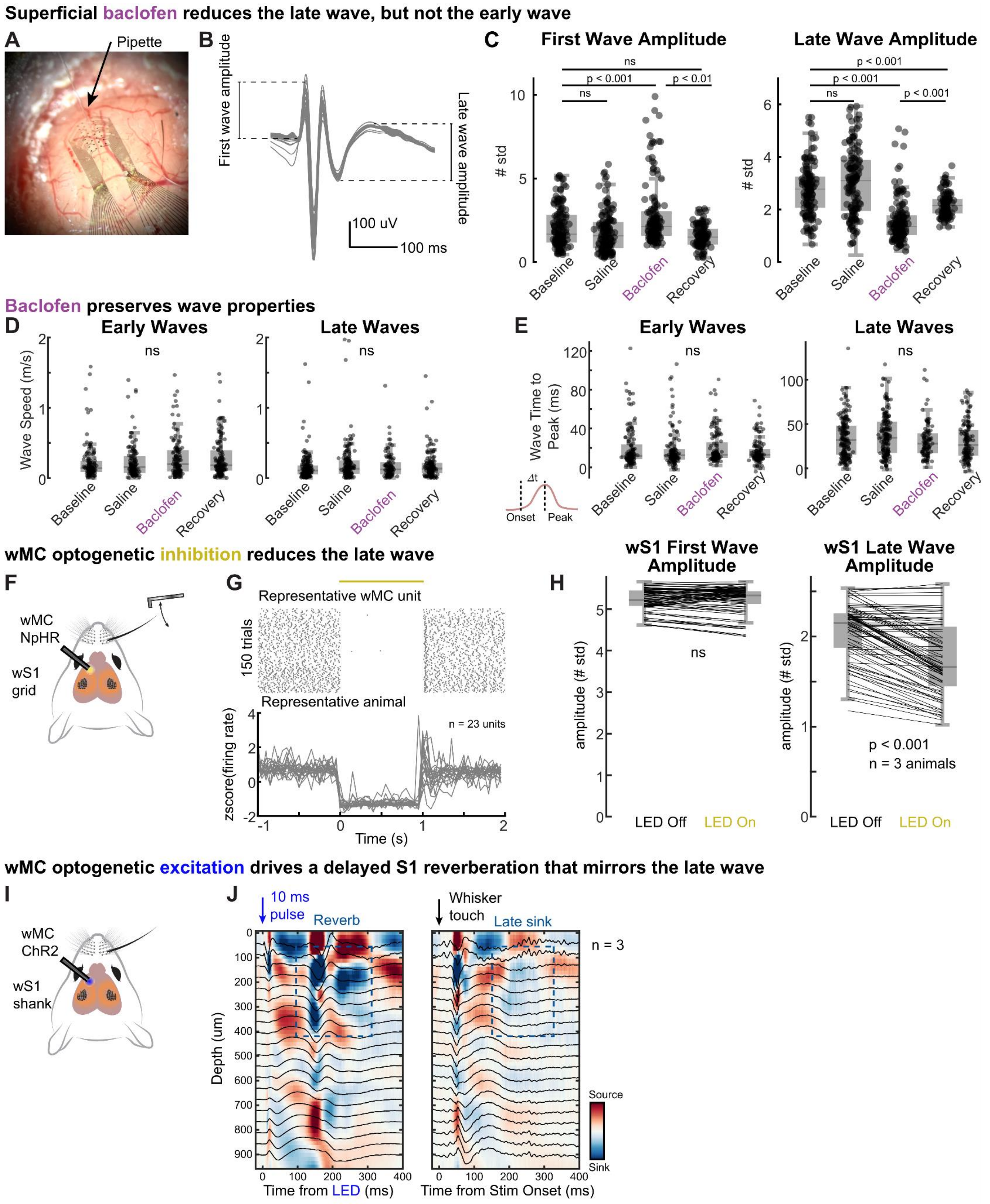

Superficial baclofen reduces the late wave, but not the early wave
A
Pipette
B
First wave amplitude
Late wave amplitude
100 uV
100 ms
C
First Wave Amplitude
Late Wave Amplitude
ns
p < 0.001
p < 0.01
p < 0.001
std
Baseline
Saline
Baclofen
Recovery
Baclofen preserves wave properties
D
Early Waves
Late Waves
Wave Speed (m/s)
E
Wave Time to Peak (ms)
Δt
Onset
Peak
wMC optogenetic inhibition reduces the late wave
F
wMC NpHR
wS1 grid
G
Representative wMC unit
150 trials
Representative animal
n = 23 units
zscore(firing rate)
Time (s)
H
wS1 First Wave Amplitude
wS1 Late Wave Amplitude
amplitude (# std)
p < 0.001
n = 3 animals
LED Off
LED On
wMC optogenetic excitation drives a delayed S1 reverberation that mirrors the late wave
I
wMC ChR2
wS1 shank
J
10 ms pulse
Reverb
Whisker touch
Late sink
n = 3
Depth (um)
Source
Sink
Time from LED (ms)
Time from Stim Onset (ms)

Figure S13.

Figure S13. Extended results from wS1 baclofen injections and M1 optogenetics

(A) Image showing a puff micropipette inserted into a through-hole for pharmacology directly under the NeuroGrids.

(B) Schematic indicating how the first and late wave amplitudes were calculated from the touch-evoked LFP.

(C) The amplitude of the first wave and late wave during baseline recordings (no injections), saline injections, baclofen injections, and the recovery period. Baclofen slightly increased the first wave amplitude but dramatically decreased the late wave amplitude (n = 5 animals, Friedman test with a *post-hoc* Dunn-Sidak test). Each data point indicates the average amplitude for a NeuroGrid electrode.

(D) Early and late wave speed during baclofen injections. (n = 5 animals, baseline early/late waves n = 132/172, saline early/late waves n = 120/168, baclofen early/late waves n = 120/97, recover early/late waves n = 131/131. ns = not significant, Kruskal-Wallis test with a *post-hoc* Dunn-Sidak test).

(E) Early and late wave time-to-peak during baclofen injections. (n = 5 animals, baseline early/late waves n = 132/172, saline early/late waves n = 120/168, baclofen early/late waves n = 120/97, recover early/late waves n = 131/131).

(F) We optogenetically inhibited wMC with NpHR during whisker touch while recording wS1 LFP with the NeuroGrids.

(G) (Top) Representative wMC spike raster during optogenetic inhibition. (Bottom) Average firing rates across 23 wMC single units during inhibition. These measurements were conducted in the absence of whisker touch.

(H) Early wave and late wave amplitude (same quantification as (B)) during whisker touch with and without wMC optogenetic inhibition (n = 3 animals, ns = not significant, $p < 0.001$, signed-rank Wilcoxon test). Each data point corresponds to a NeuroGrid electrode.

(I) We optogenetically stimulated wMC with ChR2 while recording the effects of the stimulation in wS1 with a silicon probe.

(J) Average wS1 LFP and corresponding CSD profile during optogenetic stimulation (left) and whisker touch (right) in the same animal. wMC optogenetic activation drives a delayed L2/3 current sink at ~200 ms, much in the same way that whisker touch does.
